# Microtremor datasets at liquefaction site of Petobo, Central Sulawesi-Indonesia

**DOI:** 10.1016/j.dib.2020.105554

**Published:** 2020-04-18

**Authors:** Alfiansyah Yulianur, Taufiq Saidi, Bambang Setiawan, Sugianto Sugianto, Muhammad Rusdi, Muzailin Affan

**Affiliations:** a Department of Civil Engineering, Faculty of Engineering, Universitas Syiah Kuala, Indonesia; b Program Study of Geological Engineering, Faculty of Engineering, Universitas Syiah Kuala, Indonesia; c Department of Soil Science, Faculty of Agriculture, Universitas Syiah Kuala, Indonesia; d Department of Informatics, Faculty of Mathematics and Sciences, Universitas Syiah Kuala, Indonesia

**Keywords:** Microtremor, Liquefaction site, H/V, Spectral ratio, Petobo, Central sulawesi

## Abstract

The liquefaction at Petobo, Central Sulawesi affected at least 1.8 square kilometers, destroyed up to 744 houses, and caused more than 104 casualties. The data offered in this article are microtremor measurement datasets at the liquefaction site of Petobo, Central Sulawesi, Indonesia. The datasets were recorded using 3 component (3C) Guralp 6T-D broadband seismometer. There are 14 microtremor datasets, which consist of 10 datasets measured inside the liquefaction affected site and 4 datasets recorded outside the liquefaction affected site of Petobo, Central Sulawesi. Two to four datasets with 20 min length, were recorded at different times at each location. The microtremor data is crucial for horizontal to vertical (H/V) spectral ratio analysis, from which both the fundamental frequency and ellipticity curve at the measured site are deduced. The site fundamental frequency is useful for estimating the characteristics of the sub-surface condition at the measured site. The curve of the Rayleigh waves fundamental mode is needed to develop the shear wave (S-wave) velocity profile of the measured site.

Specifications Table

**Table:** 

Subject	Civil Engineering and Geophysics
Specific subject area	Geotechnical Earthquake Engineering, Geotechnical Engineering, Near-surface geophysics
Type of data	Raw, Table, Image, Figure
How data were acquired	The raw microtremor data were collected using 3 components (3C) Guralp CMG 6T-D broadband seismometer with an integrated digitizer.
	The analyzed data were produced by authors using the Geopsy software package.
Data format	Raw and Analysed
Parameters for data collection	Raw microtremor data were collected using a sample rate of 100 samples per second. The used parameters in analyzed data included the smoothing type of Konno & Omachi with a smoothing constant of 40, a width of 5% window Tukey type, and output frequency from 0.5 to 15 Hz. All the data were collected from 19 to 21 December 2018.
Description of data collection	Onsite/field conditions were recorded during the raw data collection. Any potential disturbances to the raw data i.e. near traffic, industrial noise sources, wind, rain, and the pedestrian were recorded in the field datasheet. The raw microtremor data were collected by placing a triaxial sensor seismometer in a stable ground at the targeted location. The used seismometer is integrated with a built-in data recorder. A GPS antenna was connected to the seismometer during data measurement. A portable computer was employed for initial set up and check. A small portable battery was used to generate the system power. The seismometer was set over a generally firm to stiff ground surface. The ground, where the seismometer was fixed, was cleared of any roots, pebbles, tall grass and, then, leveled to stabilize the seismometer during data recording. The north-oriented seismometer was, also, protected from the wind and rain with a cover of a plastic bucket and stabilized with the debris of brick or concrete slab. In the present paper, at least one hour of microtremor data is considered enough for representing the measured location.
Data source location	City/Town/Region: Palu, Central Sulawesi
	Country: Indonesia
	Latitude and longitude (and GPS coordinates) for collected samples/data: [see Appendix A of the Supplementary Data associated with this paper]
Data accessibility	With the article and in the following repository data.
	Repository name: Mendeley Data
	Data identification number: DOI: 10.17632/thpdf5vp8p.1
	Direct URL to data: https://data.mendeley.com/datasets/thpdf5vp8p/1

## Value of the data

• The microtremor datasets can be used to enhance further tests at other liquefaction cases.
• The microtremor datasets can be compared and related to different in-situ measurement tools or methods to provide a greater understanding of the liquefaction evaluation.
• The H/V spectral ratio ellipticity curves can be enhanced by other researchers i.e. geophysics, geotechnical engineer, seismologist for further experiments
• The datasets are significant to assess and validate the reliability of microtremor data analysis at the liquefaction site.

## 1 Data description

**Fig. 1:**
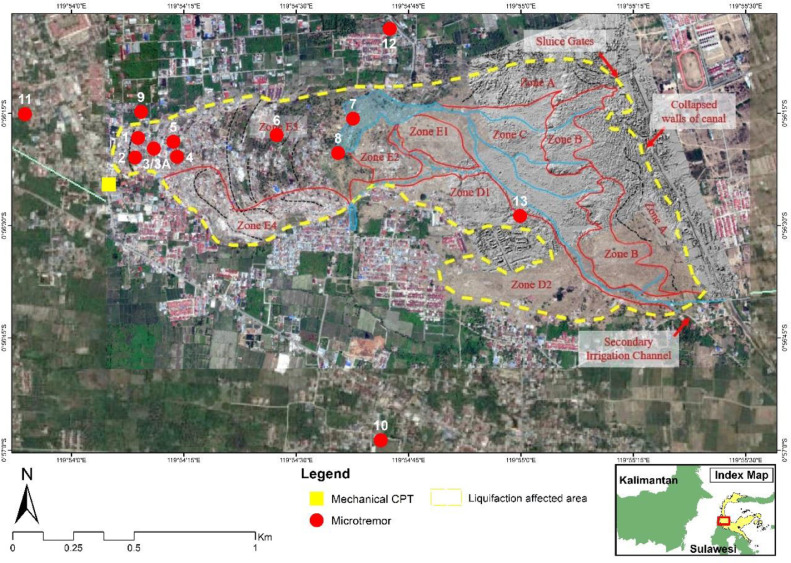
The plotted locations of the recorded microtremor data incorporated with mechanical cone penetration test (m-CPT) in [8].

On 28 September 2018, there was amateur video evidence of liquefaction triggered by an earthquake at Petobo, Central Sulawesi, Indonesia. The liquefaction at Petobo affected at least 1.8 square kilometers (Fig. 1), destroyed up to 744 houses, and caused more than 104 casualties. This liquefaction was caused by an earthquake with a magnitude of 7.5 struck north of the liquefaction area on the same date at 6:03 pm Indonesian local time (10:03 am UTC).

**Fig. 2:**
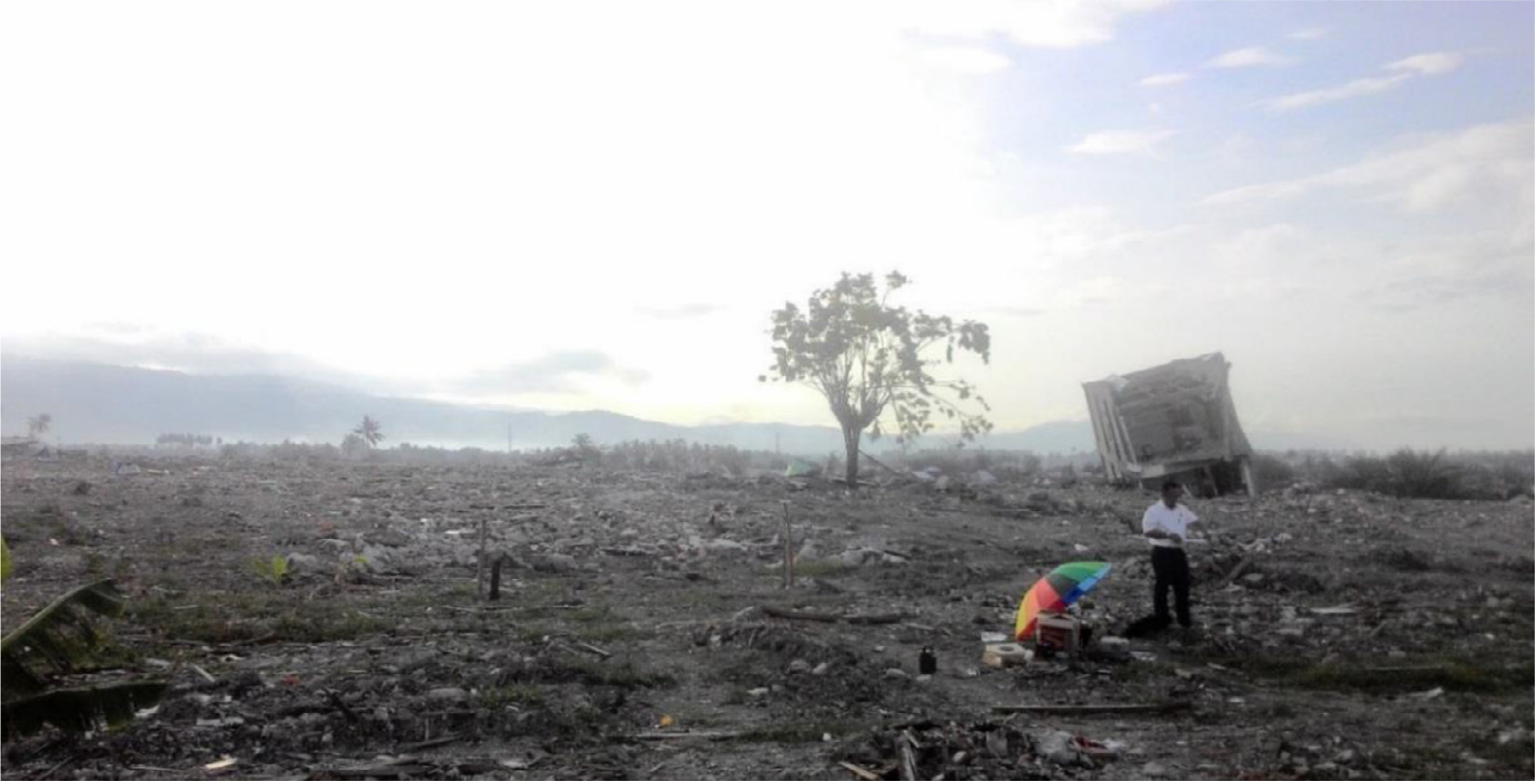
Field site conditions during the microtremor measurement.

**Fig. 3:**
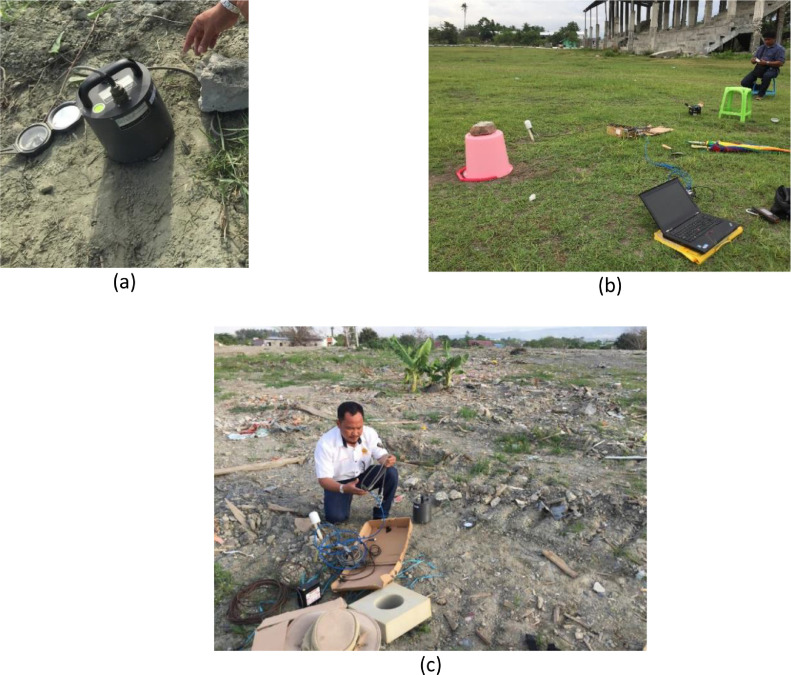
(a) Guralp seismometer; (b) recording process; and (c) setting up activity at a location.

Microtremor measurement (Fig. 2) is useful in investigating the near-surface sub-structure for seismic hazard assessment (cf. [1], [2], [3], [4], [5]). Currently, there is a consensus among the experts on the sources and nature of the noise-field as shown in [6]. This microtremor is one of the most common passive seismic methods used in urban environments as it is low-cost and efficient [7]. A broadband seismometer (Fig. 3) was deployed to the liquefaction site of Petobo, Central Sulawesi. Fourteen locations were measured, 10 inside the liquefaction zone and 4 outside the liquefaction zone, as shown in red circles in Fig. 1.

**Fig. 4:**
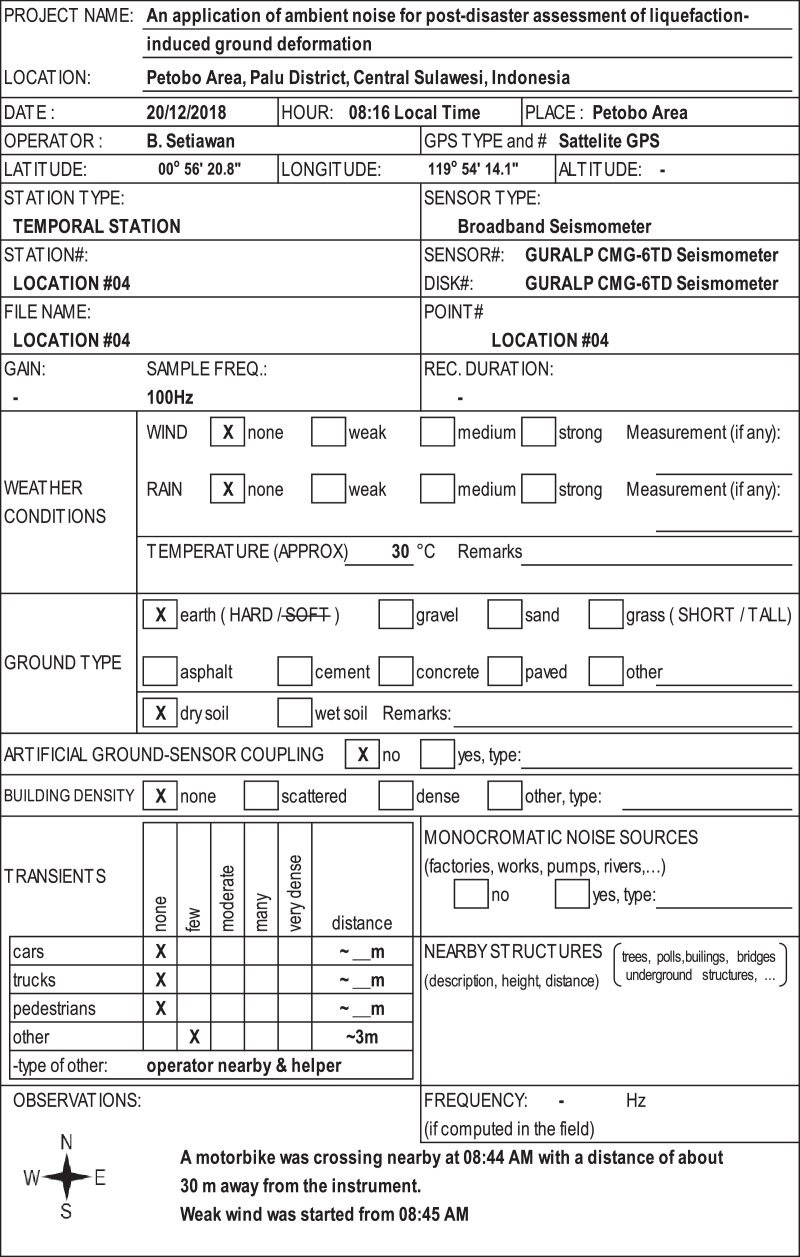
One of field datasheets.

Onsite/field conditions of the measured location were recorded during the raw data collection. Any events and potential disturbances to the recorded data i.e. near traffic, industrial noise sources, wind, rain, and a pedestrian were recorded in the field datasheet. One of the field data sheets is presented in Fig. 4. All field recorded data sheets are in Appendix A of the Supplementary Data associated with this paper.

**Fig. 5:**
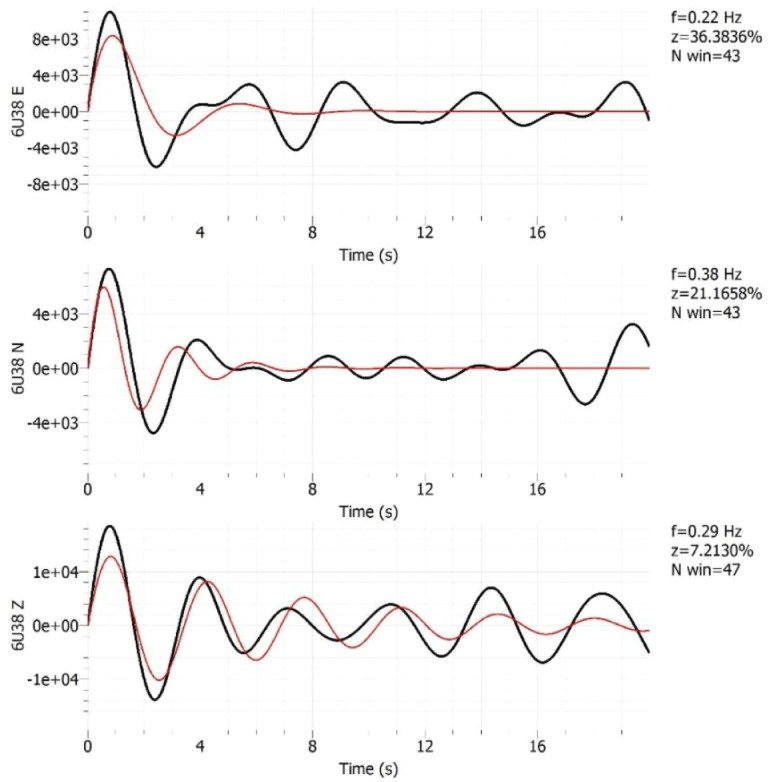
One of the damping tests for the three components (*Z* > 1% in the three components).

Before producing the analyzed data format, a damping toolbox of Geospsy [9] is employed to detect the presence of any raw data generating from any industrial sources. This detection is justifying the validity of the recorded microtremor data used in producing the analyzed data format. Some typical damping detection tests are shown in Fig. 5. All the damping data are attached in Appendix B.

**Fig. 6:**
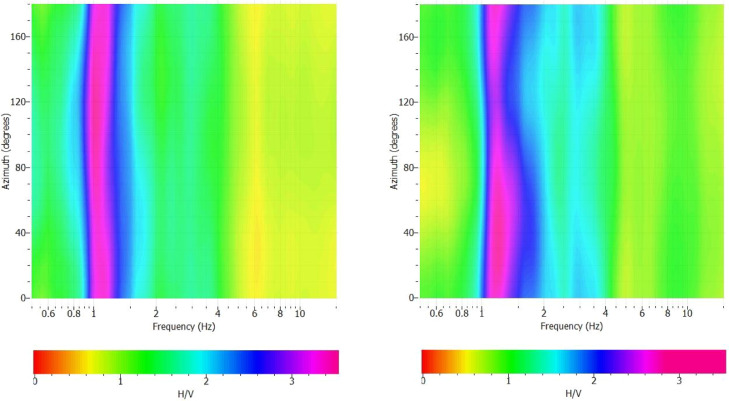
Typical H/V rotation at two measured sites of #01 (left) and #04 (right).

Fig. 6 is presented to provide more information about the H/V spectral ratio. Fig. 6 demonstrates a shaded contour map of the microtremor H/V spectral ratio as a function of the direction of ground motion and frequency. Fig. 6 is constructed by rotating the recorded motions at a location into a specific azimuth. The H/V spectral ratio was, then, computed for this rotating motion for a range of azimuths from 0° to 180°, clockwise from north. Appendix C presents the directional H/V spectra as a function of frequency and azimuth. The 0° and 90° directions show the North and the East, respectively.

**Fig. 7:**
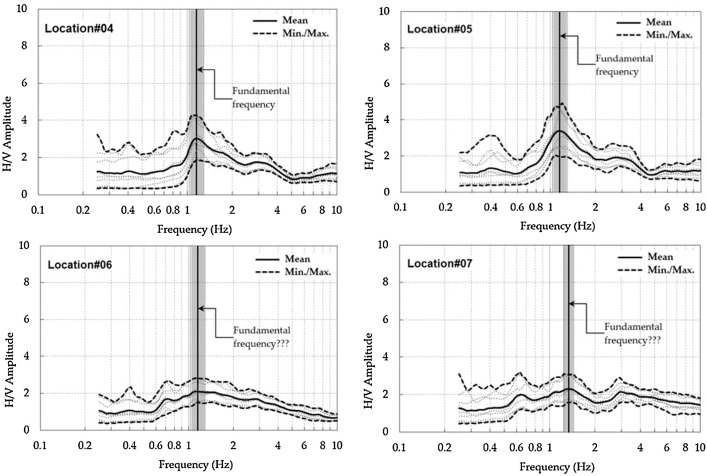
Selected H/V spectral ratio curves.

The H/V curves of Fig. 7 and Appendix D are deduced from the spectral ratio analysis between the horizontal (H) and vertical (V) components Fourier amplitude spectra of the raw data. To remove transient waves, i.e. footsteps, nearby traffic, the most stationary parts of the microtremor data were selected by dividing the time series of each microtremor component into windows. This initial step was carried out by using window length, the short-time average (STA)/long-time average (LTA) and lengths of STA/LTA. Then, Konno & Ohmachi [10] smoothing constant of 40 was employed in each selected window. Tukey window type with a width of 5% was selected. Geopsy software package [9] was used to produce the H/V spectral ratio curve at output frequency from 0.5 to 15 Hz.

## 2 Experimental design, materials, and methods

The present paper focuses on microtremor measurement at the liquefaction site of Petobo, Central Sulawesi, Indonesia (Fig. 1). In this respect, the main aim of the present paper is to provide the microtremor datasets and developed shear wave (S-wave) velocity (Vs) profiles solely from single microtremor analysis at the aforementioned, liquefaction site of Petobo.

To collect the data, a seismometer was deployed to the liquefaction site of Petobo, as shown in Fig. 2. The deployed seismometer recorded the 3 axes of microtremor waves: two horizontal components (i.e. east-west, north-south) and one vertical component (up-down). The recorded microtremor data were saved to an internal memory storage within the instrument.

The data was collected using the Guralp CMG-6TD seismometer (Fig. 3a). This seismometer is an ultra-lightweight digital three-axis seismometer in a sealed case. Thus, this seismometer can measure the 3 axes of microtremor vibrations over a wide frequency range of 0.033 to 50 Hertz simultaneously. In this Guralp CMG-6TD, an integrated 24-bit digitizer converts ground motions to digital data and save it into an internal hard disk.

As demonstrated in Fig. 3a, the used seismometer has a rugged, water-resistant design for ease of setting up. In the field, generally, this Guralp CMG-6TD seismometer requires a little adjustment for leveling. Once it is provided with 12 V battery power (in this measurement) the Guralp 6TD was operating, recording and digitizing the ground movements, and saving them into internal flash memory (Fig. 3b) automatically. Fig. 3c shows three main components used equipment in this measurement, which are the seismometer, the GPS antenna (for accurate timing), and the battery power supply. These three devices are connected through a breakout box using cables.

At least an hour noise measurement was recorded at every point. A sampling frequency of 100 Hz was set in the seismometer. The field conditions during the measurement were logged in a field data sheet [6,11]. The incorporated damping toolbox in Geopsy [10] was used in the pre-processing analysis of industrial ground motion sources. Generally, the presence of this industrial vibration source is determined if both the damping is below 1% and the frequency is constant.

The experimental horizontal-vertical (H/V) spectral ratio is a technique to evaluate the dynamic characteristics of the measured site i.e. fundamental frequency, S-wave velocity profile. As aforementioned, this technique is an analysis of the spectral ratio of the horizontal (H) vibration Fourier amplitude spectrum over vertical (V) vibration Fourier amplitude spectrum of the recorded microtremor.

A process by which the reliability of the H/V spectral ratio curve can be evaluated using SESAME [6] criteria, as follows: (i) the fundamental frequency of *f_0_* must be greater than 10 divided by the window length of *I_w_*, which is 40 s in this paper, for the peak to be significant; (ii) the significant cycles number must be greater than 200; and (iii) the HVSR curve standard deviation amplitude, at a range frequency of 0.5*f_0_* to 2*f_0_*, must be less than two when *f_0_* > 0.5 Hz, or < 3 when *f_0_* < 0.5 Hz. All these three HVSR reliable criteria must be fulfilled before further analyses (see SESAME [6]).

In practice, the correlation between the H/V spectral ratio peak(s) and the resonance frequency of the S-wave has been outlined in [6], as follows:

• In the case of a high impedance contrast site, the H/V ratio of a body wave-field always suggests a peak around the fundamental S-wave frequency.
• In the case of laterally stratified sub-surface, the H/V ratio shows peaks at the S-wave harmonics.
• In the case of laterally stratified sub-surface with high impedance contrast, the amplitude of the first H/V peak is likely to be well correlated with the S-wave amplification.

The above findings are very useful for interpreting the experimental H/V peaks from seismic noise recordings. The experimental H/V peaks are clear for horizontally layered media with large impedance contrasts (> 4–5) and become more unclear in the case of decreasing the impedance contrasts [6]. The experimental H/V peaks also become uncertain in the case of increasing underground interface slopes. Therefore, gathering the available representative geological and geotechnical information to assist in interpreting the H/V peaks in particular estimations of impedance contrasts are suggested.

## Appendix Supplementary materials

Image, application 1

Image, application 2

Image, application 3

Image, application 4
