# Supplementary material for: Microtremor datasets at liquefaction site of Petobo, Central Sulawesi-Indonesia
Source: Data Brief. 2020 Apr 18;30:105554. doi: 10.1016/j.dib.2020.105554 (PMC7184131; doi:10.1016/j.dib.2020.105554)
Supplement: Supplementary file 1 [file mmc1.pdf]

PROJECT NAME:	**An application of ambient noise for post-disaster assessment of liquefaction-induced ground deformation**
LOCATION:	**Petobo Area, Palu District, Central Sulawesi, Indonesia**

DATE :	**19/12/2018**	HOUR:	**11:20 Local Time**	PLACE :	**Petobo Area**
OPERATOR :	**B. Setiawan & T. Saidi**			GPS TYPE and #	**Sattelite GPS**
LATITUDE:	**00° 56' 18.33"**	LONGITUDE:	**119° 54' 08.9"**	ALTITUDE:	**-**

STATION TYPE: **TEMPORAL STATION**	SENSOR TYPE: **Broadband Seismometer**
STATION#: **LOCATION #01**	SENSOR#: **GURALP CMG-6TD Seismometer**<br>DISK#: **GURALP CMG-6TD Seismometer**
FILE NAME: **LOCATION #01**	POINT# **LOCATION #01**
GAIN: **-** SAMPLE FREQ.: **100Hz**	REC. DURATION: **At least an hour**

WEATHER CONDITIONS

WIND [ ] none [ ] weak [X] medium [ ] strong Measurement (if any): ______

RAIN [X] none [ ] weak [ ] medium [ ] strong Measurement (if any): ______

TEMPERATURE (APPROX) **32** °C Remarks ______

GROUND TYPE

[X] earth (HARD / ~~SOFT~~) [ ] gravel [ ] sand [ ] grass (SHORT / TALL)

[ ] asphalt [ ] cement [ ] concrete [ ] paved [ ] other ______

[X] dry soil [ ] wet soil Remarks: ______

ARTIFICIAL GROUND-SENSOR COUPLING [X] no [ ] yes, type: ______

BUILDING DENSITY [X] none [ ] scattered [ ] dense [ ] other, type: ______

TRANSIENTS

	none	few	moderate	many	very dense	distance
cars	X					~ __m
trucks	X					~ __m
pedestrians		X				~30m
other						
-type of other:	**occasionally people nearby**					

MONOCROMATIC NOISE SOURCES (factories, works, pumps, rivers,…)

[ ] no [ ] yes, type: ______

NEARBY STRUCTURES (description, height, distance) { trees, polls,builings, bridges underground structures, ... }

OBSERVATIONS:

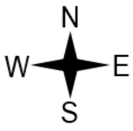

The 1st 30 minutes is without bucket cover.

FREQUENCY: **-** Hz (if computed in the field)

PROJECT NAME:	**An application of ambient noise for post-disaster assessment of liquefaction-induced ground deformation**
LOCATION:	**Petobo Area, Palu District, Central Sulawesi, Indonesia**

DATE : **19/12/2018**	HOUR: **12:38 Local Time**	PLACE : **Petobo Area**	
OPERATOR : **B. Setiawan & T. Saidi**		GPS TYPE and # **Sattelite GPS**	
LATITUDE: **00° 56' 20.9"**	LONGITUDE: **119° 54' 08.5"**	ALTITUDE: **-**	

STATION TYPE: **TEMPORAL STATION**	SENSOR TYPE: **Broadband Seismometer**
STATION#: **LOCATION #02**	SENSOR#: **GURALP CMG-6TD Seismometer**<br>DISK#: **GURALP CMG-6TD Seismometer**
FILE NAME: **LOCATION #02**	POINT# **LOCATION #02**
GAIN: **-** / SAMPLE FREQ.: **100Hz**	REC. DURATION: **At least an hour**

WEATHER CONDITIONS

WIND [ ] none [ ] weak [X] medium [ ] strong Measurement (if any): ______

RAIN [X] none [ ] weak [ ] medium [ ] strong Measurement (if any): ______

TEMPERATURE (APPROX) **33** °C Remarks ______

GROUND TYPE

[X] earth (HARD / ~~SOFT~~) [ ] gravel [ ] sand [ ] grass (SHORT / TALL)

[ ] asphalt [ ] cement [ ] concrete [ ] paved [ ] other ______

[X] dry soil [ ] wet soil Remarks: ______

ARTIFICIAL GROUND-SENSOR COUPLING [X] no [ ] yes, type: ______

BUILDING DENSITY [X] none [ ] scattered [ ] dense [ ] other, type: ______

TRANSIENTS

	none	few	moderate	many	very dense	distance
cars	X					~ __m
trucks	X					~ __m
pedestrians	X					~ __m
other						~3m
-type of other:	**operator nearby**					

MONOCROMATIC NOISE SOURCES (factories, works, pumps, rivers,…) [ ] no [ ] yes, type: ______

NEARBY STRUCTURES (description, height, distance) { trees, polls,builings, bridges underground structures, ... }

OBSERVATIONS:

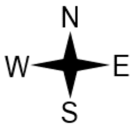

FREQUENCY: **-** Hz (if computed in the field)

PROJECT NAME:	**An application of ambient noise for post-disaster assessment of liquefaction-induced ground deformation**
LOCATION:	**Petobo Area, Palu District, Central Sulawesi, Indonesia**

DATE :	**19/12/2018**	HOUR:	**13:56 Local Time**	PLACE :	**Petobo Area**
OPERATOR :	**B. Setiawan & T. Saidi**	GPS TYPE and #	**Sattelite GPS**		
LATITUDE:	**00° 56' 19.7"**	LONGITUDE:	**119° 54' 11.0"**	ALTITUDE:	**-**

STATION TYPE: **TEMPORAL STATION**	SENSOR TYPE: **Broadband Seismometer**
STATION#: **LOCATION #03**	SENSOR#: **GURALP CMG-6TD Seismometer**<br>DISK#: **GURALP CMG-6TD Seismometer**
FILE NAME: **LOCATION #03**	POINT# **LOCATION #03**
GAIN: **-** SAMPLE FREQ.: **100Hz**	REC. DURATION: **-**

WEATHER CONDITIONS

WIND: [ ] none [ ] weak [X] medium [X] strong Measurement (if any): ____

RAIN: [X] none [ ] weak [ ] medium [ ] strong Measurement (if any): ____

TEMPERATURE (APPROX) **33** °C Remarks ____

GROUND TYPE

[X] earth (HARD / ~~SOFT~~) [ ] gravel [ ] sand [ ] grass (SHORT / TALL)

[ ] asphalt [ ] cement [ ] concrete [ ] paved [ ] other ____

[X] dry soil [ ] wet soil Remarks: ____

ARTIFICIAL GROUND-SENSOR COUPLING [X] no [ ] yes, type: ____

BUILDING DENSITY [X] none [ ] scattered [ ] dense [ ] other, type: ____

TRANSIENTS

	none	few	moderate	many	very dense	distance
cars	X					~ __m
trucks	X					~ __m
pedestrians	X					~ __m
other		X				~3m
-type of other:	**operator nearby & helper**					

MONOCROMATIC NOISE SOURCES (factories, works, pumps, rivers,...) [ ] no [ ] yes, type: ____

NEARBY STRUCTURES (description, height, distance) { trees, polls,builings, bridges underground structures, ... }

FREQUENCY: **-** Hz (if computed in the field)

OBSERVATIONS:

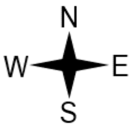

The recording was stopped due to rain.

PROJECT NAME:	**An application of ambient noise for post-disaster assessment of liquefaction-induced ground deformation**		
LOCATION:	**Petobo Area, Palu District, Central Sulawesi, Indonesia**		
DATE : **20/12/2018**	HOUR: **07:10 Local Time**	PLACE : **Petobo Area**	
OPERATOR : **B. Setiawan & T. Saidi**		GPS TYPE and # **Sattelite GPS**	
LATITUDE: **00° 56' 19.7"**	LONGITUDE: **119° 54' 11.0"**	ALTITUDE: **-**	
STATION TYPE: **TEMPORAL STATION**		SENSOR TYPE: **Broadband Seismometer**	
STATION#: **LOCATION #03A**		SENSOR#: **GURALP CMG-6TD Seismometer**<br>DISK#: **GURALP CMG-6TD Seismometer**	
FILE NAME: **LOCATION #03A**		POINT# **LOCATION #03A**	
GAIN: **-**	SAMPLE FREQ.: **100Hz**	REC. DURATION: **-**	

WEATHER CONDITIONS

- WIND: [X] none [ ] weak [ ] medium [ ] strong — Measurement (if any): ____
- RAIN: [X] none [ ] weak [ ] medium [ ] strong — Measurement (if any): ____
- TEMPERATURE (APPROX) **25** °C — Remarks ____

GROUND TYPE

- [X] earth (HARD / ~~SOFT~~) [ ] gravel [ ] sand [ ] grass (SHORT / TALL)
- [ ] asphalt [ ] cement [ ] concrete [ ] paved [ ] other ____
- [X] dry soil [ ] wet soil — Remarks: ____

ARTIFICIAL GROUND-SENSOR COUPLING [X] no [ ] yes, type: ____

BUILDING DENSITY [X] none [ ] scattered [ ] dense [ ] other, type: ____

TRANSIENTS

	none	few	moderate	many	very dense	distance
cars	X					**~ __m**
trucks	X					**~ __m**
pedestrians	X					**~ __m**
other		X				**~3m**
-type of other:	**operator nearby & helper**					

MONOCROMATIC NOISE SOURCES (factories, works, pumps, rivers,...) [ ] no [ ] yes, type: ____

NEARBY STRUCTURES (description, height, distance) { trees, polls,builings, bridges underground structures, ... }

OBSERVATIONS: Flights was take off at:
-07:20 AM
-07:30 AM
-07:37 AM
-07:47 AM

Airport is about 2 km away

N / W / E / S

FREQUENCY: **-** Hz (if computed in the field)

PROJECT NAME:	**An application of ambient noise for post-disaster assessment of liquefaction-induced ground deformation**
LOCATION:	**Petobo Area, Palu District, Central Sulawesi, Indonesia**

DATE :	**20/12/2018**	HOUR:	**08:16 Local Time**	PLACE :	**Petobo Area**
OPERATOR :	**B. Setiawan**			GPS TYPE and #	**Sattelite GPS**
LATITUDE:	**00° 56' 20.8"**	LONGITUDE:	**119° 54' 14.1"**	ALTITUDE:	**-**

STATION TYPE: **TEMPORAL STATION**	SENSOR TYPE: **Broadband Seismometer**
STATION#: **LOCATION #04**	SENSOR#: **GURALP CMG-6TD Seismometer**<br>DISK#: **GURALP CMG-6TD Seismometer**
FILE NAME: **LOCATION #04**	POINT# **LOCATION #04**
GAIN: **-** SAMPLE FREQ.: **100Hz**	REC. DURATION: **-**

WEATHER CONDITIONS

WIND [X] none [ ] weak [ ] medium [ ] strong Measurement (if any): ____

RAIN [X] none [ ] weak [ ] medium [ ] strong Measurement (if any): ____

TEMPERATURE (APPROX) **30** °C Remarks ____

GROUND TYPE

[X] earth (HARD / ~~SOFT~~) [ ] gravel [ ] sand [ ] grass (SHORT / TALL)

[ ] asphalt [ ] cement [ ] concrete [ ] paved [ ] other ____

[X] dry soil [ ] wet soil Remarks: ____

ARTIFICIAL GROUND-SENSOR COUPLING [X] no [ ] yes, type: ____

BUILDING DENSITY [X] none [ ] scattered [ ] dense [ ] other, type: ____

TRANSIENTS

	none	few	moderate	many	very dense	distance
cars	**X**					**~ __m**
trucks	**X**					**~ __m**
pedestrians	**X**					**~ __m**
other		**X**				**~3m**
-type of other:	**operator nearby & helper**					

MONOCROMATIC NOISE SOURCES (factories, works, pumps, rivers,...)
[ ] no [ ] yes, type: ____

NEARBY STRUCTURES (description, height, distance) { trees, polls,builings, bridges underground structures, ... }

FREQUENCY: **-** Hz (if computed in the field)

OBSERVATIONS:

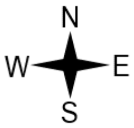

A motorbike was crossing nearby at 08:44 AM with a distance of about 30 m away from the instrument.
Weak wind was started from 08:45 AM

PROJECT NAME: **An application of ambient noise for post-disaster assessment of liquefaction-induced ground deformation**

LOCATION: **Petobo Area, Palu District, Central Sulawesi, Indonesia**

DATE :	**20/12/2018**	HOUR:	**09:38 Local Time**	PLACE :	**Petobo Area**
OPERATOR :	**B. Setiawan**		GPS TYPE and #	**Sattelite GPS**	
LATITUDE:	**00° 56' 18.8"**	LONGITUDE:	**119° 54' 13.6"**	ALTITUDE:	**-**

STATION TYPE: **TEMPORAL STATION**	SENSOR TYPE: **Broadband Seismometer**
STATION#: **LOCATION #05**	SENSOR#: **GURALP CMG-6TD Seismometer**<br>DISK#: **GURALP CMG-6TD Seismometer**
FILE NAME: **LOCATION #05**	POINT# **LOCATION #05**
GAIN: **-** SAMPLE FREQ.: **100Hz**	REC. DURATION: **-**

WEATHER CONDITIONS

WIND [X] none [X] weak [ ] medium [ ] strong Measurement (if any): ______

RAIN [X] none [ ] weak [ ] medium [ ] strong Measurement (if any): ______

TEMPERATURE (APPROX) **30** °C Remarks ______

GROUND TYPE

[X] earth (HARD / ~~SOFT~~) [ ] gravel [ ] sand [ ] grass (SHORT / TALL)

[ ] asphalt [ ] cement [ ] concrete [ ] paved [ ] other ______

[X] dry soil [ ] wet soil Remarks: ______

ARTIFICIAL GROUND-SENSOR COUPLING [X] no [ ] yes, type: ______

BUILDING DENSITY [X] none [ ] scattered [ ] dense [ ] other, type: ______

TRANSIENTS

	none	few	moderate	many	very dense	distance
cars	**X**					**~ __m**
trucks	**X**					**~ __m**
pedestrians	**X**					**~ __m**
other		**X**				**~3m**
-type of other:	**operator nearby & helper**					

MONOCROMATIC NOISE SOURCES (factories, works, pumps, rivers,…) [ ] no [ ] yes, type: ______

NEARBY STRUCTURES (description, height, distance) { trees, polls,builings, bridges underground structures, ... }

OBSERVATIONS:

FREQUENCY: **-** Hz (if computed in the field)

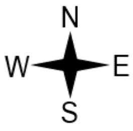

Weak wind was started from 09:50 AM

Water level is @-0.5m

PROJECT NAME:	**An application of ambient noise for post-disaster assessment of liquefaction-induced ground deformation**
LOCATION:	**Petobo Area, Palu District, Central Sulawesi, Indonesia**

DATE :	**20/12/2018**	HOUR:	**12:16 Local Time**	PLACE :	**Petobo Area**
OPERATOR :	**B. Setiawan**			GPS TYPE and #	**Sattelite GPS**
LATITUDE:	**00° 56' 17.9"**	LONGITUDE:	**119° 54' 27.4"**	ALTITUDE:	**-**

STATION TYPE: **TEMPORAL STATION**	SENSOR TYPE: **Broadband Seismometer**
STATION#: **LOCATION #06**	SENSOR#: **GURALP CMG-6TD Seismometer**<br>DISK#: **GURALP CMG-6TD Seismometer**
FILE NAME: **LOCATION #06**	POINT# **LOCATION #06**
GAIN: **-** SAMPLE FREQ.: **100Hz**	REC. DURATION: **-**

WEATHER CONDITIONS

- WIND: [ ] none [ ] weak [X] medium [X] strong Measurement (if any): ______
- RAIN: [X] none [ ] weak [ ] medium [ ] strong Measurement (if any): ______
- TEMPERATURE (APPROX) **33** °C Remarks ______

GROUND TYPE

- [X] earth (HARD / ~~SOFT~~) [ ] gravel [ ] sand [ ] grass (SHORT / TALL)
- [ ] asphalt [ ] cement [ ] concrete [ ] paved [ ] other ______
- [X] dry soil [ ] wet soil Remarks: ______

ARTIFICIAL GROUND-SENSOR COUPLING [X] no [ ] yes, type: ______

BUILDING DENSITY [X] none [ ] scattered [ ] dense [ ] other, type: ______

TRANSIENTS

	none	few	moderate	many	very dense	distance
cars	X					~ __m
trucks	X					~ __m
pedestrians	X					~ __m
other	X					~__m
-type of other:	**operator nearby & helper**					

MONOCROMATIC NOISE SOURCES (factories, works, pumps, rivers,…)
[ ] no [ ] yes, type: ______

NEARBY STRUCTURES (description, height, distance) (trees, polls,builings, bridges underground structures, ...)

Coconut trees are at about 20 m away.

OBSERVATIONS:

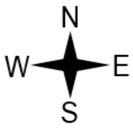

Water level is @-0.2m

FREQUENCY: **-** Hz
(if computed in the field)

PROJECT NAME: **An application of ambient noise for post-disaster assessment of liquefaction-induced ground deformation**

LOCATION: **Petobo Area, Palu District, Central Sulawesi, Indonesia**

DATE :	**20/12/2018**	HOUR:	**13:40 Local Time**	PLACE :	**Petobo Area**
OPERATOR :	**B. Setiawan**	GPS TYPE and #	**Sattelite GPS**		
LATITUDE:	**00° 56' 15.7"**	LONGITUDE:	**119° 54' 37.6"**	ALTITUDE:	**-**

STATION TYPE: **TEMPORAL STATION**	SENSOR TYPE: **Broadband Seismometer**
STATION#: **LOCATION #07**	SENSOR#: **GURALP CMG-6TD Seismometer**<br>DISK#: **GURALP CMG-6TD Seismometer**
FILE NAME: **LOCATION #07**	POINT# **LOCATION #07**
GAIN: **-** SAMPLE FREQ.: **100Hz**	REC. DURATION: **-**

WEATHER CONDITIONS

WIND: [ ] none [ ] weak [X] medium [X] strong Measurement (if any): ____

RAIN: [X] none [ ] weak [ ] medium [ ] strong Measurement (if any): ____

TEMPERATURE (APPROX) **34** °C Remarks ____

GROUND TYPE

[X] earth (HARD / ~~SOFT~~) [ ] gravel [X] sand [ ] grass (SHORT / TALL)

[ ] asphalt [ ] cement [ ] concrete [ ] paved [ ] other ____

[X] dry soil [ ] wet soil Remarks: ____

ARTIFICIAL GROUND-SENSOR COUPLING [X] no [ ] yes, type: ____

BUILDING DENSITY [X] none [ ] scattered [ ] dense [ ] other, type: ____

TRANSIENTS

	none	few	moderate	many	very dense	distance
cars	X					~ __m
trucks	X					~ __m
pedestrians	X					~ __m
other	X					~__m
-type of other:	**operator nearby & helper**					

MONOCROMATIC NOISE SOURCES (factories, works, pumps, rivers,…) [ ] no [ ] yes, type: ____

NEARBY STRUCTURES (description, height, distance) { trees, polls,builings, bridges underground structures, ... }

OBSERVATIONS:

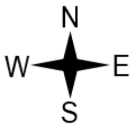

Water level is @-0.3m

FREQUENCY: **-** Hz (if computed in the field)

PROJECT NAME:	**An application of ambient noise for post-disaster assessment of liquefaction-induced ground deformation**		
LOCATION:	**Petobo Area, Palu District, Central Sulawesi, Indonesia**		
DATE : **20/12/2018**	HOUR: **14:50 Local Time**	PLACE : **Petobo Area**	
OPERATOR : **B. Setiawan**		GPS TYPE and # **Sattelite GPS**	
LATITUDE: **00° 56' 20.3"**	LONGITUDE: **119° 54' 35.6"**	ALTITUDE: **-**	
STATION TYPE: **TEMPORAL STATION**		SENSOR TYPE: **Broadband Seismometer**	
STATION#: **LOCATION #08**		SENSOR#: **GURALP CMG-6TD Seismometer**<br>DISK#: **GURALP CMG-6TD Seismometer**	
FILE NAME: **LOCATION #08**		POINT# **LOCATION #08**	
GAIN: **-**	SAMPLE FREQ.: **100Hz**	REC. DURATION: **-**	

WEATHER CONDITIONS

- WIND: [ ] none [ ] weak [X] medium [X] strong — Measurement (if any): ______
- RAIN: [X] none [ ] weak [ ] medium [ ] strong — Measurement (if any): ______
- TEMPERATURE (APPROX) **32** °C — Remarks ______

GROUND TYPE

- [X] earth (HARD / ~~SOFT~~) [ ] gravel [X] sand [ ] grass (SHORT / TALL)
- [ ] asphalt [ ] cement [ ] concrete [ ] paved [ ] other ______
- [X] dry soil [ ] wet soil — Remarks: ______

ARTIFICIAL GROUND-SENSOR COUPLING [X] no [ ] yes, type: ______

BUILDING DENSITY [X] none [ ] scattered [ ] dense [ ] other, type: ______

TRANSIENTS

	none	few	moderate	many	very dense	distance
cars	X					~ __m
trucks	X					~ __m
pedestrians	X					~ __m
other	X					~__m
-type of other:	**operator nearby & helper**					

MONOCROMATIC NOISE SOURCES (factories, works, pumps, rivers,...) [ ] no [ ] yes, type: ______

NEARBY STRUCTURES (description, height, distance) (trees, polls,builings, bridges underground structures, ...)

FREQUENCY: **-** Hz (if computed in the field)

OBSERVATIONS:

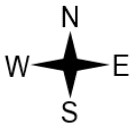

Airplanes were landed @15:10 pm and 15:40pm.

PROJECT NAME:	**An application of ambient noise for post-disaster assessment of liquefaction-induced ground deformation**
LOCATION:	**Petobo Area, Palu District, Central Sulawesi, Indonesia**

DATE :	**20/12/2018**	HOUR:	**16:35 Local Time**	PLACE :	**Petobo Area**
OPERATOR :	**B. Setiawan**			GPS TYPE and #	**Sattelite GPS**
LATITUDE:	**00° 56' 14.8"**	LONGITUDE:	**119° 54' 09.3"**	ALTITUDE:	**-**

STATION TYPE: **TEMPORAL STATION**	SENSOR TYPE: **Broadband Seismometer**
STATION#: **LOCATION #09**	SENSOR#: **GURALP CMG-6TD Seismometer**<br>DISK#: **GURALP CMG-6TD Seismometer**
FILE NAME: **LOCATION #09**	POINT# **LOCATION #09**
GAIN: **-** SAMPLE FREQ.: **100Hz**	REC. DURATION: **-**

WEATHER CONDITIONS

- WIND: [X] none [X] weak [ ] medium [ ] strong Measurement (if any): ____
- RAIN: [X] none [ ] weak [ ] medium [ ] strong Measurement (if any): ____
- TEMPERATURE (APPROX) **30** °C Remarks ____

GROUND TYPE

- [ ] earth (HARD / ~~SOFT~~) [ ] gravel [ ] sand [ ] grass (SHORT / TALL)
- [ ] asphalt [X] cement [ ] concrete [ ] paved [ ] other ____
- [ ] dry soil [ ] wet soil Remarks: ____

ARTIFICIAL GROUND-SENSOR COUPLING [X] no [ ] yes, type: ____

BUILDING DENSITY [ ] none [X] scattered [ ] dense [ ] other, type: ____

TRANSIENTS

	none	few	moderate	many	very dense	distance
cars	X					~ __m
trucks	X					~ __m
pedestrians	X					~ __m
other	X					~__m
-type of other:	**operator nearby & helper**					

MONOCROMATIC NOISE SOURCES (factories, works, pumps, rivers,...) [ ] no [ ] yes, type: ____

NEARBY STRUCTURES (description, height, distance) (trees, polls,builings, bridges underground structures, ...)

FREQUENCY: **-** Hz (if computed in the field)

OBSERVATIONS:

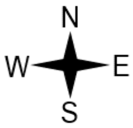

An airplanes was take off @16:40pm.
Slight rain was started from 16:45pm.

PROJECT NAME:	An application of ambient noise for post-disaster assessment of liquefaction-induced ground deformation				
LOCATION:	Petobo Area, Palu District, Central Sulawesi, Indonesia				
DATE :	21/12/2018	HOUR: 08:15 Local Time		PLACE : Petobo Area	
OPERATOR :	B. Setiawan		GPS TYPE and # Sattelite GPS		
LATITUDE:	00° 56' 58.7"	LONGITUDE:	119° 54' 41.3"	ALTITUDE: -	
STATION TYPE: TEMPORAL STATION			SENSOR TYPE: Broadband Seismometer		
STATION#: LOCATION #10			SENSOR#: GURALP CMG-6TD Seismometer<br>DISK#: GURALP CMG-6TD Seismometer		
FILE NAME: LOCATION #10			POINT# LOCATION #10		
GAIN: -	SAMPLE FREQ.: 100Hz		REC. DURATION: -		

WEATHER CONDITIONS

WIND [X] none [ ] weak [ ] medium [ ] strong Measurement (if any): ____

RAIN [X] none [ ] weak [ ] medium [ ] strong Measurement (if any): ____

TEMPERATURE (APPROX) 23 °C Remarks ____

GROUND TYPE

[X] earth (HARD / ~~SOFT~~) [ ] gravel [ ] sand [ ] grass (SHORT / TALL)

[ ] asphalt [ ] cement [ ] concrete [ ] paved [ ] other ____

[X] dry soil [ ] wet soil Remarks: ____

ARTIFICIAL GROUND-SENSOR COUPLING [X] no [ ] yes, type: ____

BUILDING DENSITY [X] none [X] scattered [ ] dense [ ] other, type: ____

TRANSIENTS

	none	few	moderate	many	very dense	distance
cars		X				~ __m
trucks		X				~ __m
pedestrians						~ __m
other		X				~__m
-type of other:	operator nearby & helper					

MONOCROMATIC NOISE SOURCES (factories, works, pumps, rivers,...)

[ ] no [ ] yes, type: ____

NEARBY STRUCTURES (description, height, distance) (trees, polls,builings, bridges underground structures, ...)

Single story houses & sport stadium

FREQUENCY: - Hz
(if computed in the field)

OBSERVATIONS:

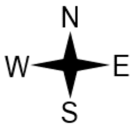

This measurement is outside the liquefaction zone.

PROJECT NAME:	An application of ambient noise for post-disaster assessment of liquefaction-induced ground deformation		
LOCATION:	Petobo Area, Palu District, Central Sulawesi, Indonesia		
DATE : 21/12/2018	HOUR: 09:20 Local Time	PLACE : Petobo Area	
OPERATOR : B. Setiawan		GPS TYPE and # Sattelite GPS	
LATITUDE: 00° 56' 15.1"	LONGITUDE: 119° 53' 53.8"	ALTITUDE: -	
STATION TYPE: TEMPORAL STATION		SENSOR TYPE: Broadband Seismometer	
STATION#: LOCATION #11		SENSOR#: GURALP CMG-6TD Seismometer<br>DISK#: GURALP CMG-6TD Seismometer	
FILE NAME: LOCATION #11		POINT# LOCATION #11	
GAIN: -	SAMPLE FREQ.: 100Hz	REC. DURATION: -	

WEATHER CONDITIONS

- WIND: [X] none [X] weak [ ] medium [ ] strong — Measurement (if any): ____
- RAIN: [X] none [ ] weak [ ] medium [ ] strong — Measurement (if any): ____
- TEMPERATURE (APPROX) 30 °C — Remarks ____

GROUND TYPE

- [X] earth (HARD / ~~SOFT~~) [X] gravel [X] sand [ ] grass (SHORT / TALL)
- [ ] asphalt [ ] cement [ ] concrete [ ] paved [ ] other ____
- [X] dry soil [ ] wet soil — Remarks: ____

ARTIFICIAL GROUND-SENSOR COUPLING: [X] no [ ] yes, type: ____

BUILDING DENSITY: [ ] none [X] scattered [ ] dense [ ] other, type: ____

TRANSIENTS

	none	few	moderate	many	very dense	distance
cars		X				~ __m
trucks	X					~ __m
pedestrians	X					~ __m
other	X					~__m
-type of other:	operator nearby & helper					

MONOCROMATIC NOISE SOURCES (factories, works, pumps, rivers,...)
[ ] no [ ] yes, type: ____

NEARBY STRUCTURES (description, height, distance) (trees, polls,builings, bridges underground structures, ...)

Single story houses are at about 15-20 m
Tall grasses are at about 5 m away.

OBSERVATIONS:

FREQUENCY: - Hz (if computed in the field)

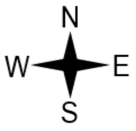

This measurement is outside the liquefaction zone.

PROJECT NAME:	**An application of ambient noise for post-disaster assessment of liquefaction-induced ground deformation**
LOCATION:	**Petobo Area, Palu District, Central Sulawesi, Indonesia**

DATE : **21/12/2018**	HOUR: **14:49 Local Time**	PLACE : **Petobo Area**	
OPERATOR : **B. Setiawan**		GPS TYPE and # **Sattelite GPS**	
LATITUDE: **00° 56' 03.7"**	LONGITUDE: **119° 54' 42.5"**	ALTITUDE: **-**	

STATION TYPE: **TEMPORAL STATION**	SENSOR TYPE: **Broadband Seismometer**
STATION#: **LOCATION #12**	SENSOR#: **GURALP CMG-6TD Seismometer**<br>DISK#: **GURALP CMG-6TD Seismometer**
FILE NAME: **LOCATION #12**	POINT# **LOCATION #12**
GAIN: **-** SAMPLE FREQ.: **100Hz**	REC. DURATION: **-**

WEATHER CONDITIONS

- WIND: [ ] none [X] weak [ ] medium [ ] strong Measurement (if any): ______
- RAIN: [X] none [ ] weak [ ] medium [ ] strong Measurement (if any): ______
- TEMPERATURE (APPROX) **32** °C Remarks ______

GROUND TYPE

- [X] earth (HARD / ~~SOFT~~) [X] gravel [X] sand [ ] grass (SHORT / TALL)
- [ ] asphalt [ ] cement [ ] concrete [ ] paved [ ] other ______
- [X] dry soil [ ] wet soil Remarks: ______

ARTIFICIAL GROUND-SENSOR COUPLING [X] no [ ] yes, type: ______

BUILDING DENSITY [ ] none [X] scattered [ ] dense [ ] other, type: ______

TRANSIENTS

	none	few	moderate	many	very dense	distance
cars		X				~ __m
trucks	X	X				~ __m
pedestrians						~ __m
other		X	X			~8m
-type of other:	operator nearby, helper, & bike					

MONOCROMATIC NOISE SOURCES (factories, works, pumps, rivers,…) [ ] no [ ] yes, type: ______

NEARBY STRUCTURES (description, height, distance) (trees, polls,builings, bridges underground structures, ...)

Single story houses are around.
Tofu machine is at 15 - 20 m away

FREQUENCY: **-** Hz (if computed in the field)

OBSERVATIONS:

N W E S

This measurement is outside the liquefaction zone.

PROJECT NAME:	**An application of ambient noise for post-disaster assessment of liquefaction-induced ground deformation**
LOCATION:	**Petobo Area, Palu District, Central Sulawesi, Indonesia**

DATE :	**21/12/2018**	HOUR:	**16:35 Local Time**	PLACE :	**Petobo Area**
OPERATOR :	**B. Setiawan**			GPS TYPE and #	**Sattelite GPS**
LATITUDE:	**00° 56' 28.7"**	LONGITUDE:	**119° 54' 59.9"**	ALTITUDE:	**-**

STATION TYPE:<br>**TEMPORAL STATION**	SENSOR TYPE:<br>**Broadband Seismometer**
STATION#:<br>**LOCATION #13**	SENSOR#: **GURALP CMG-6TD Seismometer**<br>DISK#: **GURALP CMG-6TD Seismometer**
FILE NAME:<br>**LOCATION #13**	POINT#<br>**LOCATION #13**
GAIN: **-** SAMPLE FREQ.: **100Hz**	REC. DURATION: **-**

WEATHER CONDITIONS

WIND: [ ] none [X] weak [X] medium [ ] strong Measurement (if any): ____

RAIN: [X] none [ ] weak [ ] medium [ ] strong Measurement (if any): ____

TEMPERATURE (APPROX) **30** °C Remarks ____

GROUND TYPE

[X] earth (HARD / ~~SOFT~~) [ ] gravel [ ] sand [ ] grass (SHORT / TALL)

[ ] asphalt [ ] cement [ ] concrete [ ] paved [ ] other ____

[X] dry soil [ ] wet soil Remarks: ____

ARTIFICIAL GROUND-SENSOR COUPLING [X] no [ ] yes, type: ____

BUILDING DENSITY [X] none [ ] scattered [ ] dense [ ] other, type: ____

TRANSIENTS

	none	few	moderate	many	very dense	distance
cars	**X**					**~ __m**
trucks	**X**					**~ __m**
pedestrians	**X**					**~ __m**
other		**X**				**~8m**
-type of other:	**operator nearby**					

MONOCROMATIC NOISE SOURCES (factories, works, pumps, rivers,…)
[ ] no [ ] yes, type: ____

NEARBY STRUCTURES (description, height, distance) { trees, polls,builings, bridges underground structures, ... }

FREQUENCY: **-** Hz
(if computed in the field)

OBSERVATIONS:

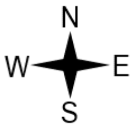

Airplanes were take off at 16:40pm and 17:05pm.
