# Supplementary material for: Microtremor datasets at liquefaction site of Petobo, Central Sulawesi-Indonesia
Source: Data Brief. 2020 Apr 18;30:105554. doi: 10.1016/j.dib.2020.105554 (PMC7184131; doi:10.1016/j.dib.2020.105554)
Supplement: Supplementary file 2 [file mmc2.pdf]

Appendix B. Results of Damping Test at All Locations

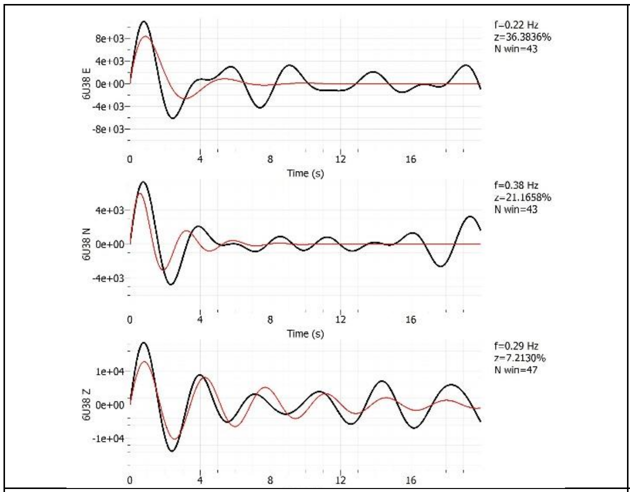

Results of Damping Test#1 at Location 01

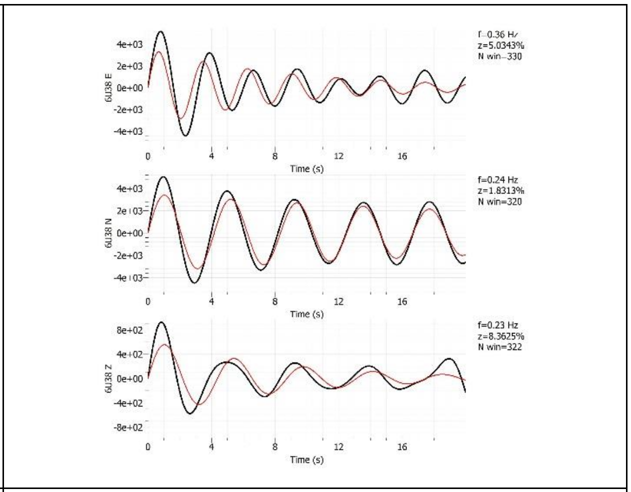

Results of Damping Test#2 at Location 01

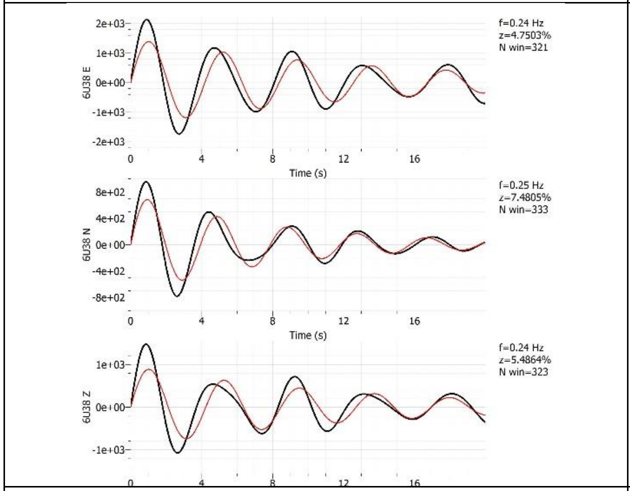

Results of Damping Test#3 at Location 01

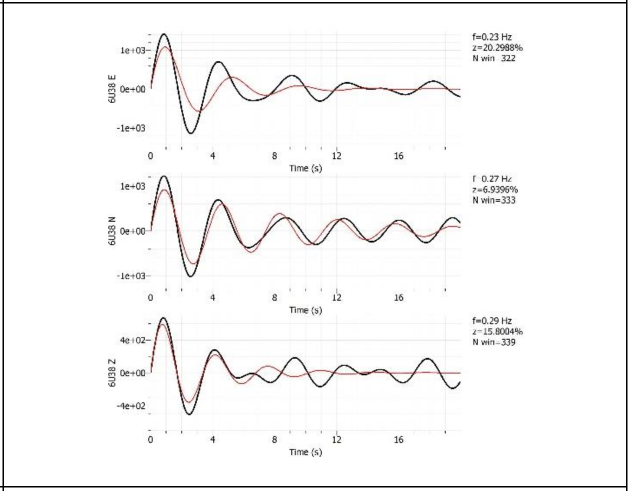

Results of Damping Test#4 at Location 01

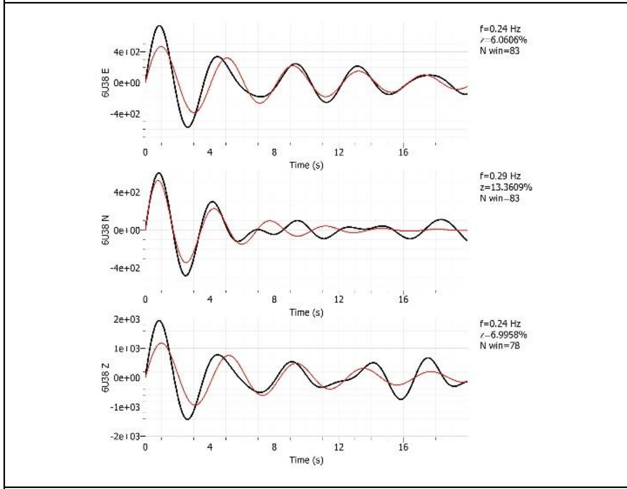

Results of Damping Test#1 at Location 02

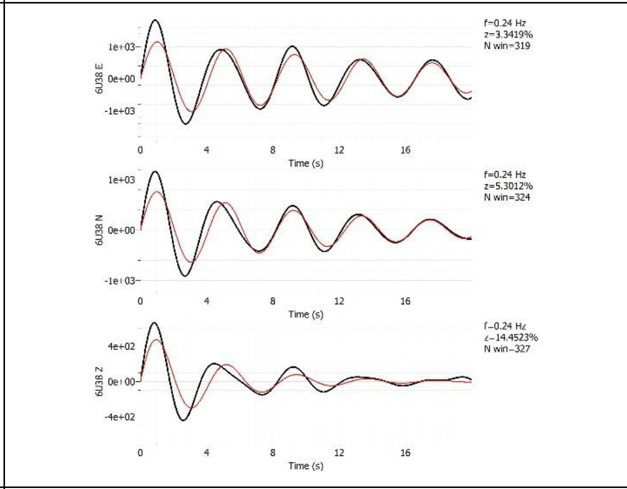

Results of Damping Test#2 at Location 02

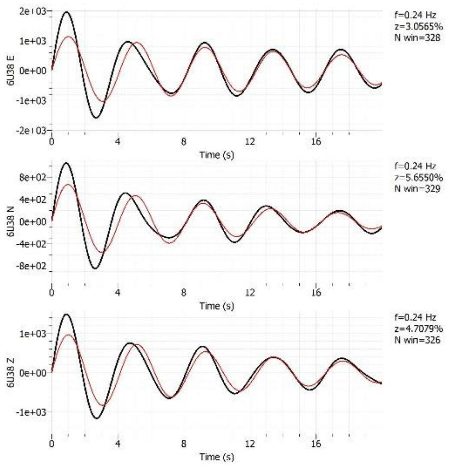

Results of Damping Test#3 at Location 02

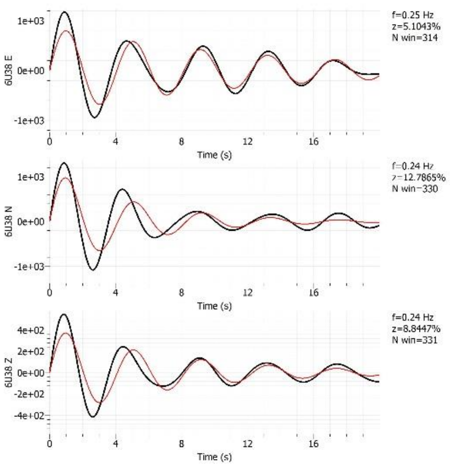

Results of Damping Test#4 at Location 02

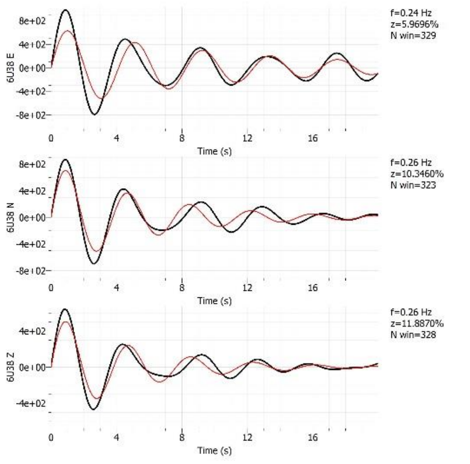

Results of Damping Test#2 at Location 03

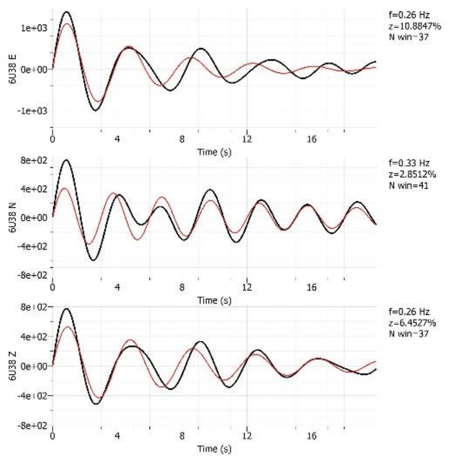

Results of Damping Test#3 at Location 03

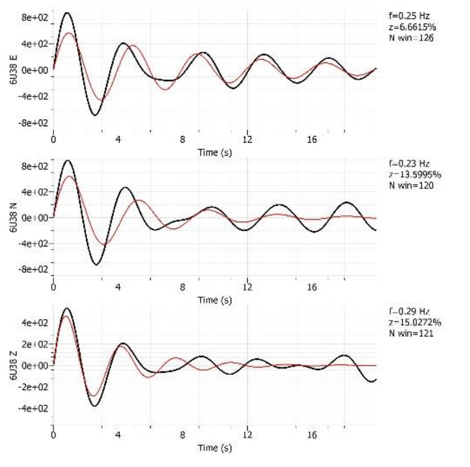

Results of Damping Test#1 at Location 03A

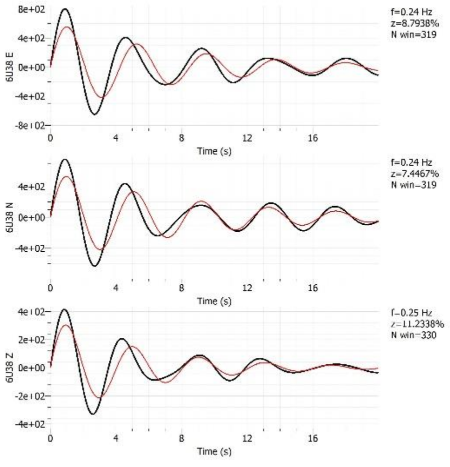

Results of Damping Test#2 at Location 03A

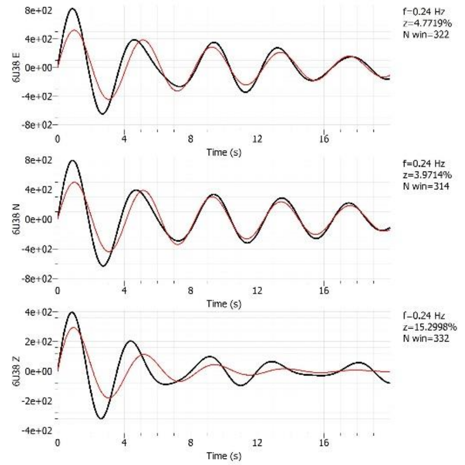

Results of Damping Test#3 at Location 03A

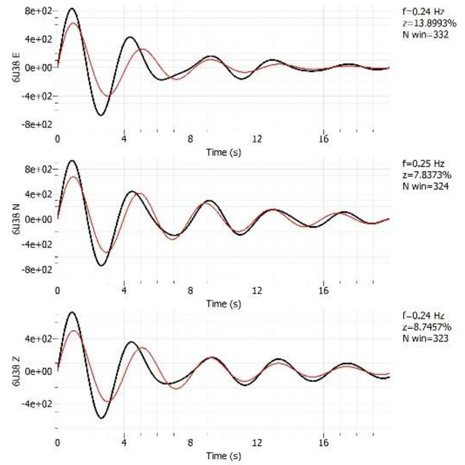

Results of Damping Test#1 at Location 04

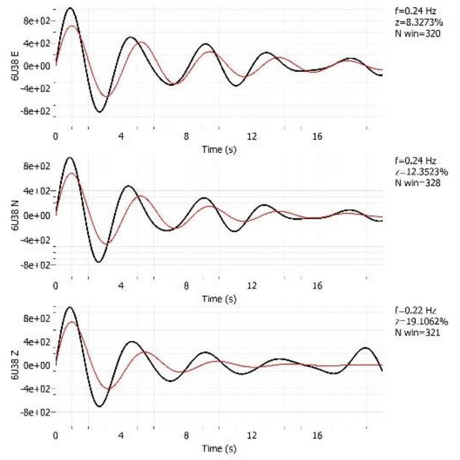

Results of Damping Test#2 at Location 04

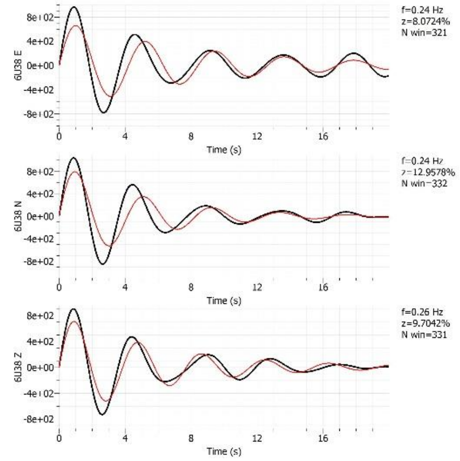

Results of Damping Test#3 at Location 04

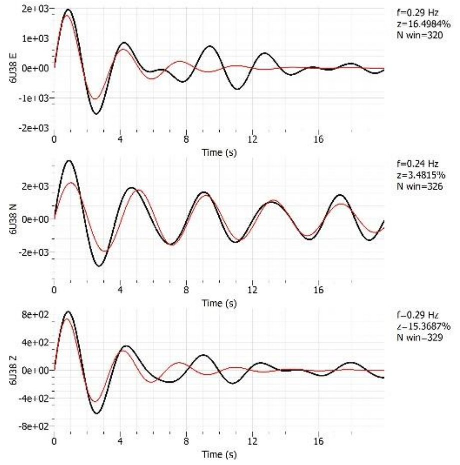

Results of Damping Test#1 at Location 05

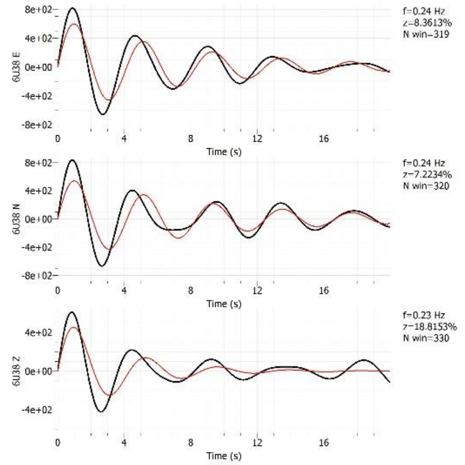

Results of Damping Test#2 at Location 05

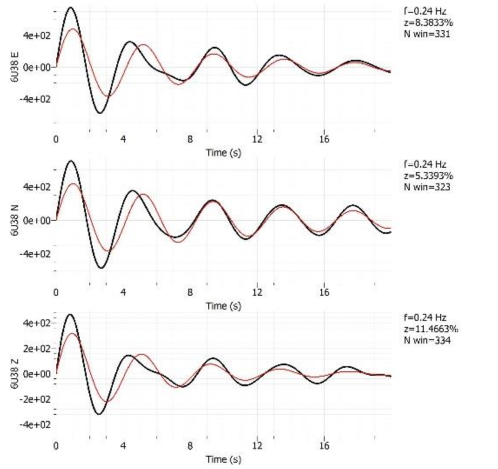

Results of Damping Test#3 at Location 05

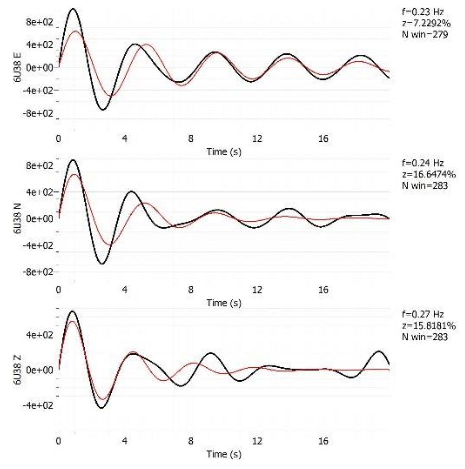

Results of Damping Test#1 at Location 06

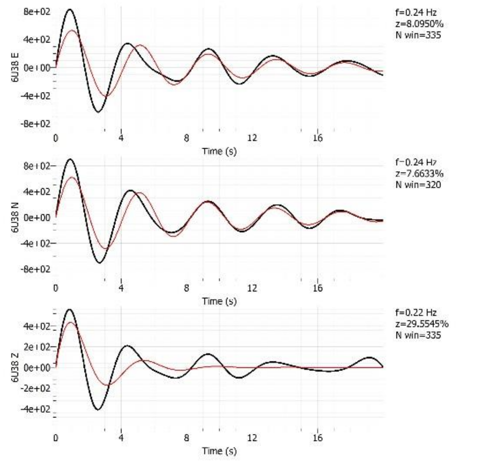

Results of Damping Test#2 at Location 06

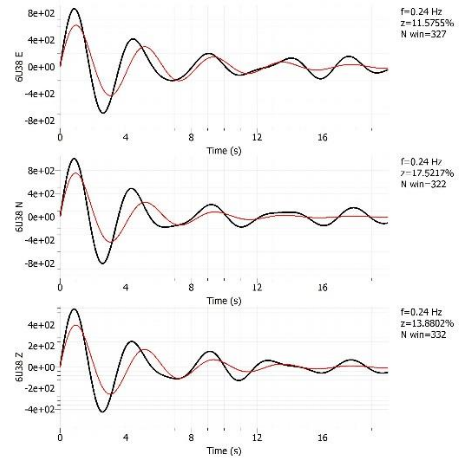

Results of Damping Test#3 at Location 06

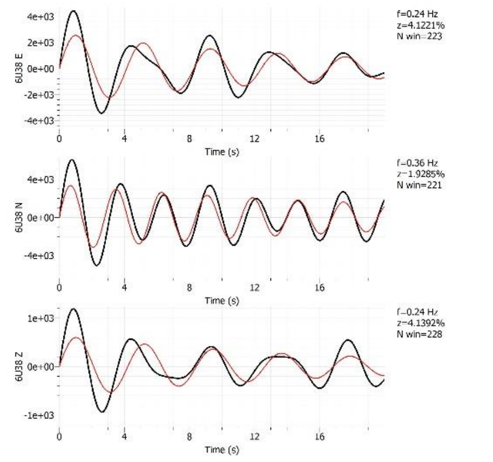

Results of Damping Test#1 at Location 07

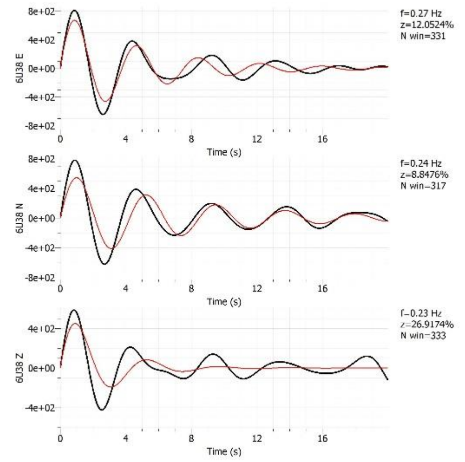

Results of Damping Test#2 at Location 07

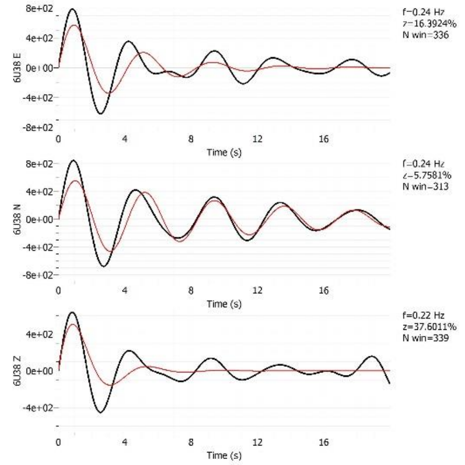

Results of Damping Test#3 at Location 07

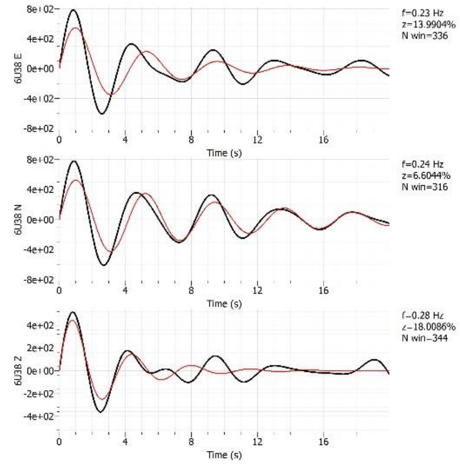

Results of Damping Test#1 at Location 08

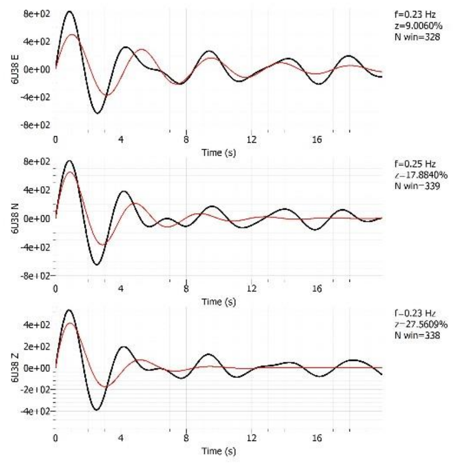

Results of Damping Test#2 at Location 08

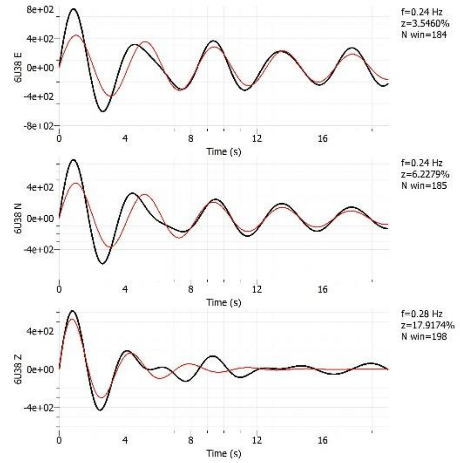

Results of Damping Test#3 at Location 08

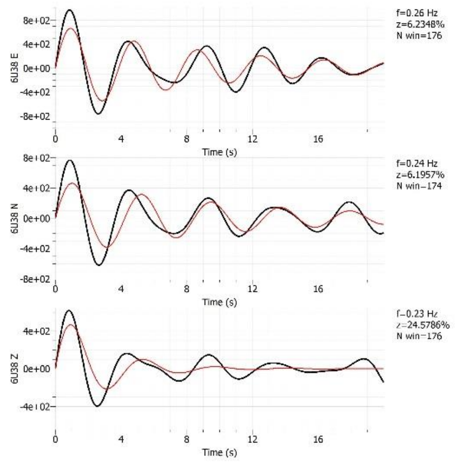

Results of Damping Test#2 at Location 09

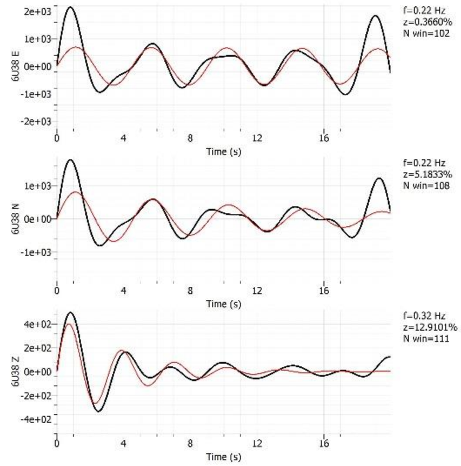

Results of Damping Test#1 at Location 10

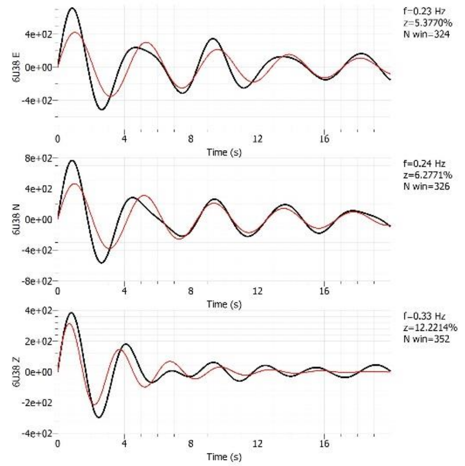

Results of Damping Test#2 at Location 10

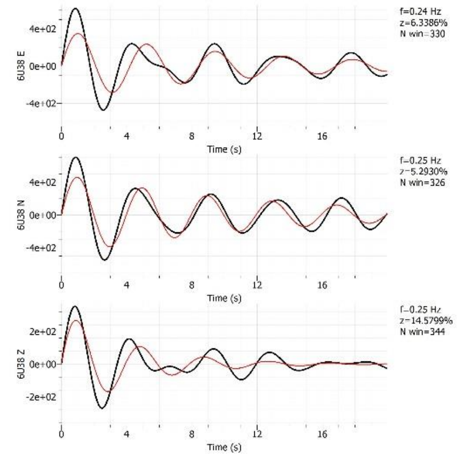

Results of Damping Test#3 at Location 10

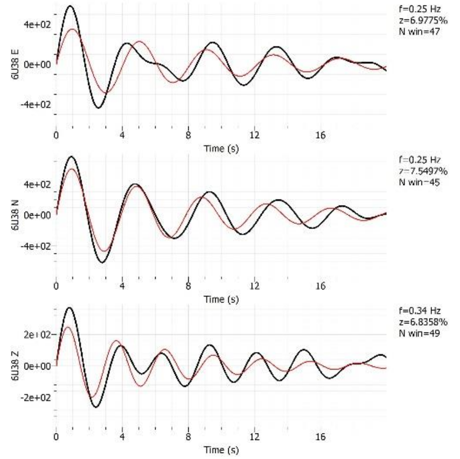

Results of Damping Test#4 at Location 10

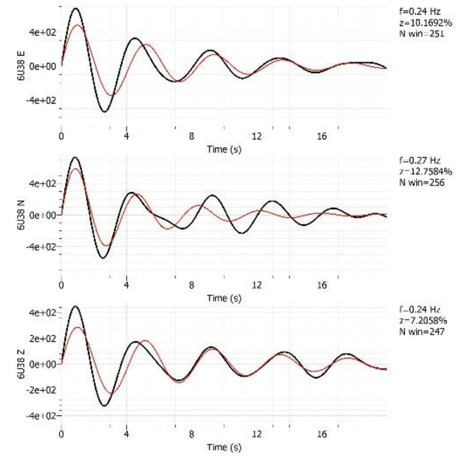

Results of Damping Test#1 at Location 11

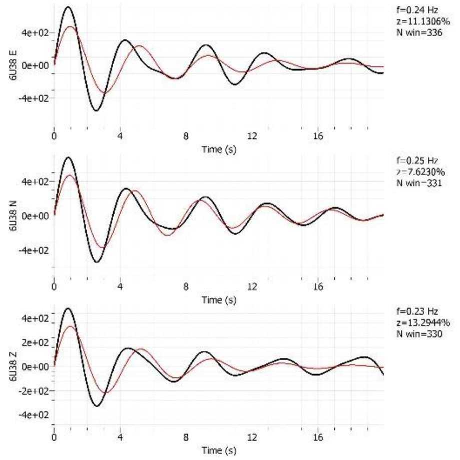

Results of Damping Test#2 at Location 11

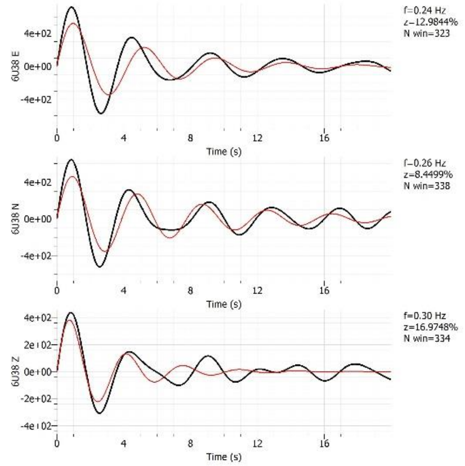

Results of Damping Test#3 at Location 11

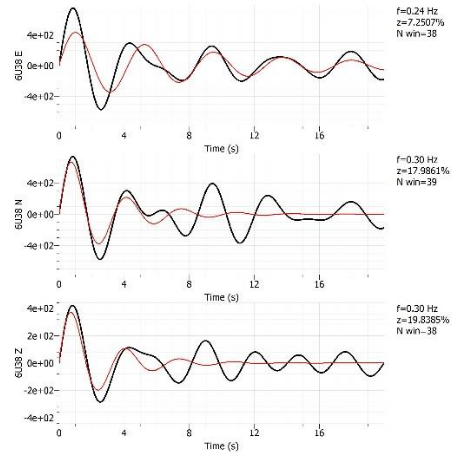	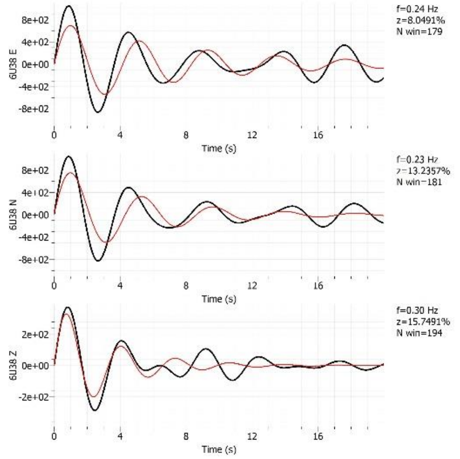
Results of Damping Test#4 at Location 11	Results of Damping Test#1 at Location 12
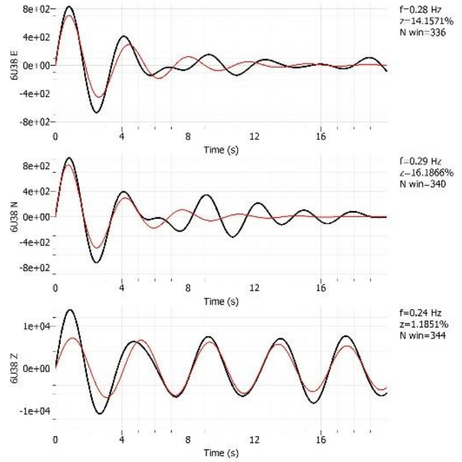	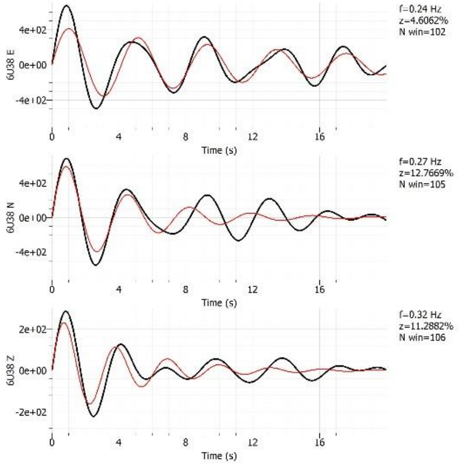
Results of Damping Test#2 at Location 12	Results of Damping Test#3 at Location 12
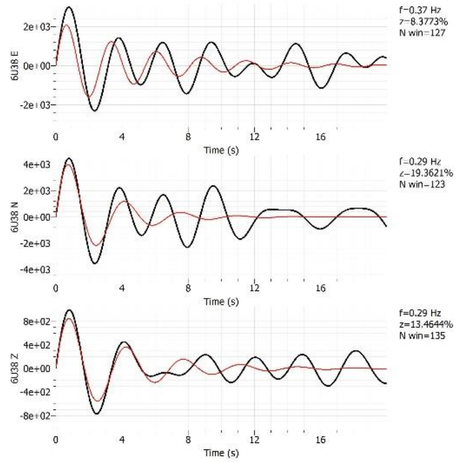	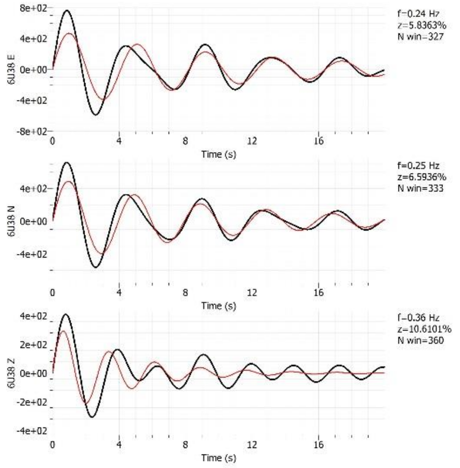
Results of Damping Test#1 at Location 13	Results of Damping Test#2 at Location 13

<table>
<tr>
<td>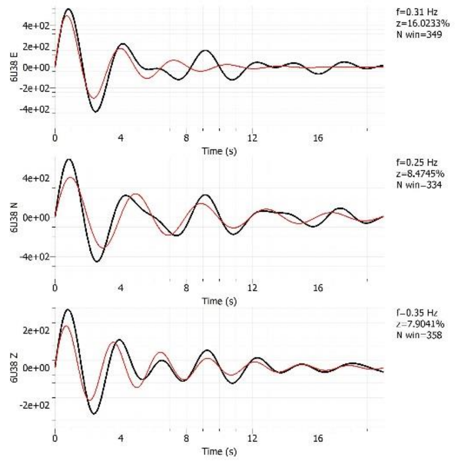
</td>
<td>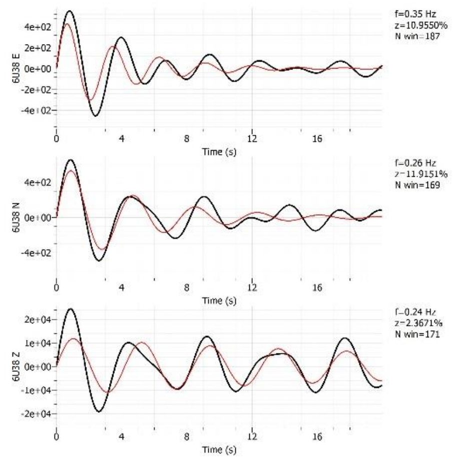
</td>
</tr>
<tr>
<td>Results of Damping Test#3 at Location 13</td>
<td>Results of Damping Test#4 at Location 13</td>
</tr>
</table>
