# Supplementary figures and images for: Microtremor datasets at liquefaction site of Petobo, Central Sulawesi-Indonesia
Source: Data Brief. 2020 Apr 18;30:105554. doi: 10.1016/j.dib.2020.105554 (PMC7184131; doi:10.1016/j.dib.2020.105554)

Appendix C. Spatial distribution of the H/V frequency results

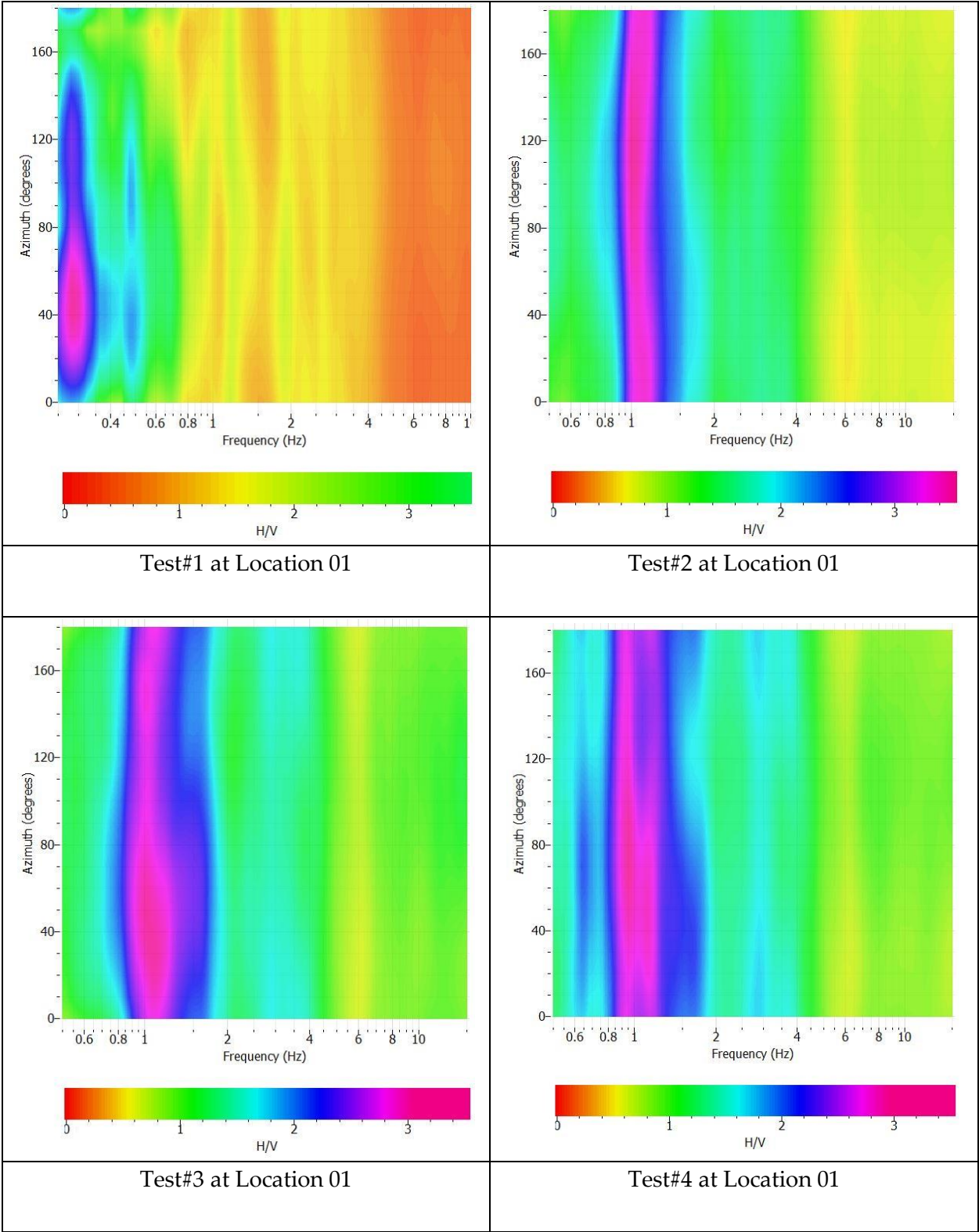

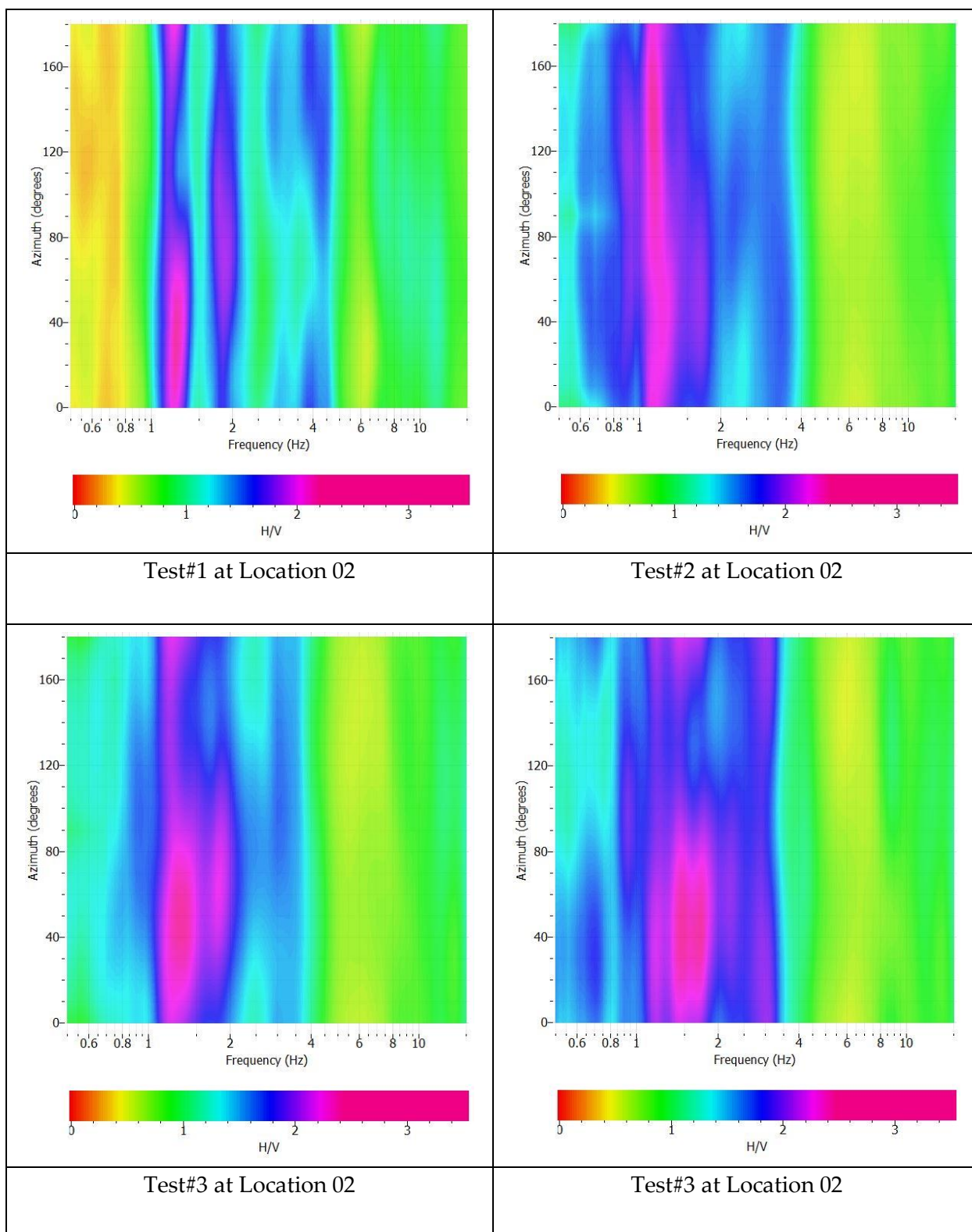

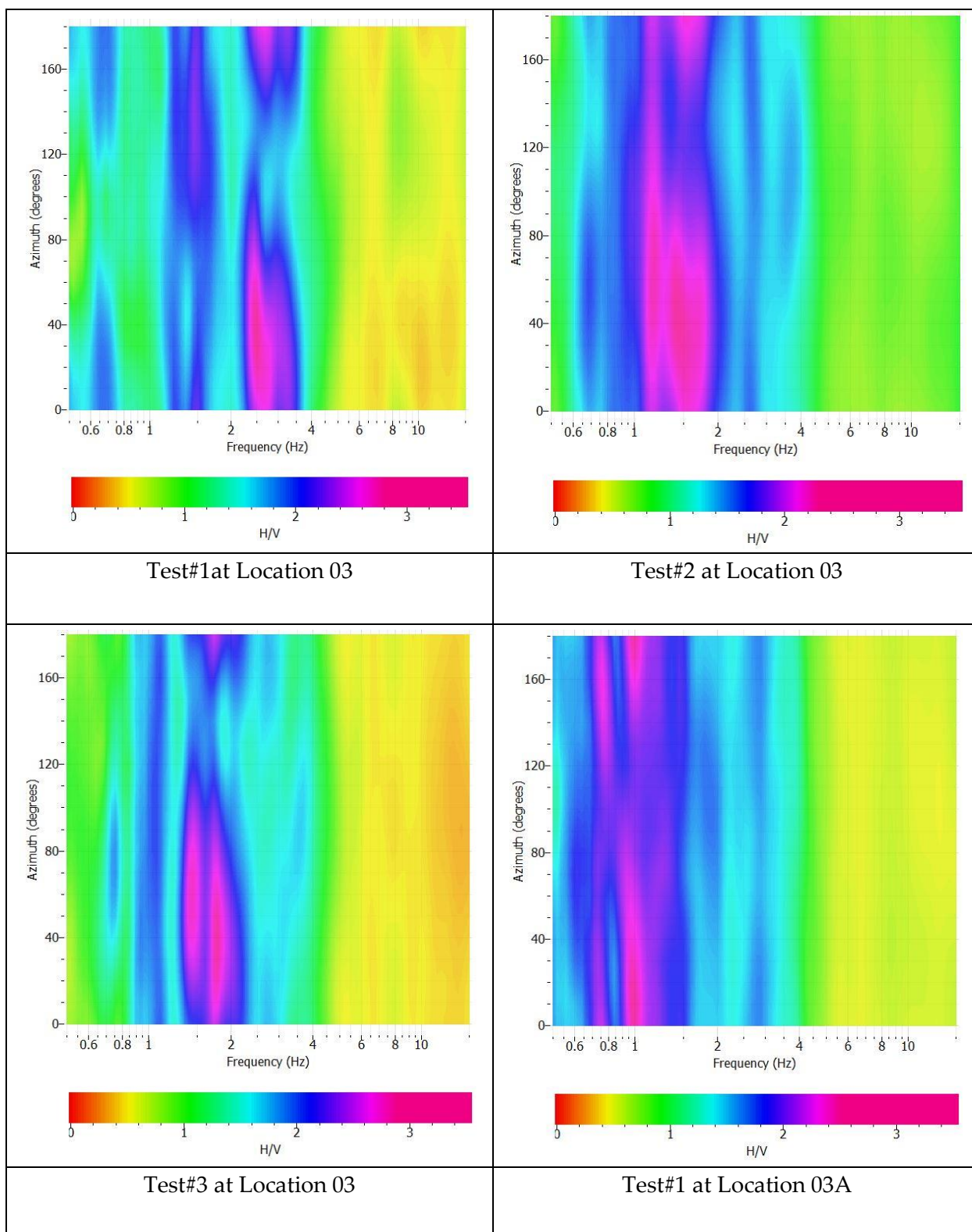

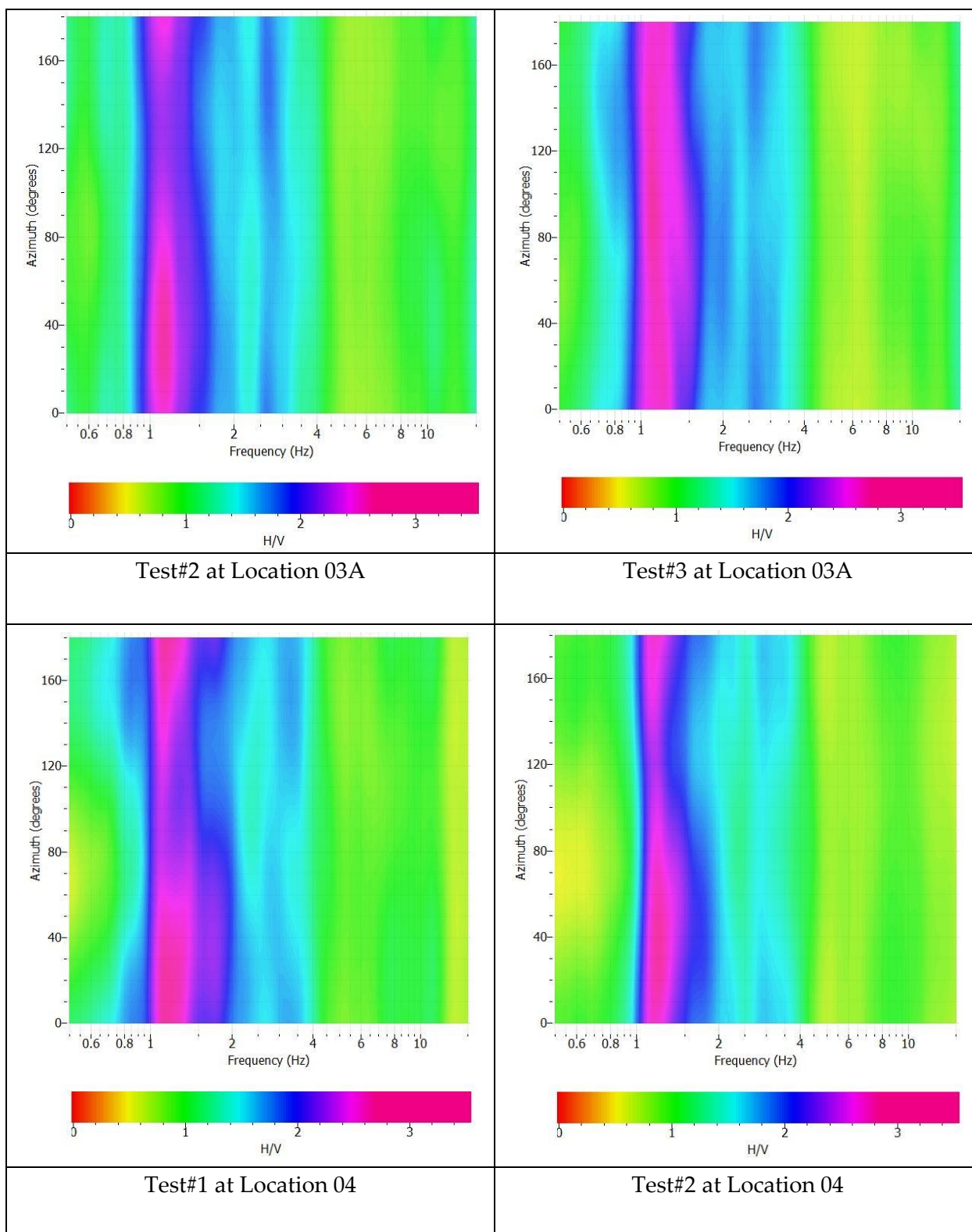

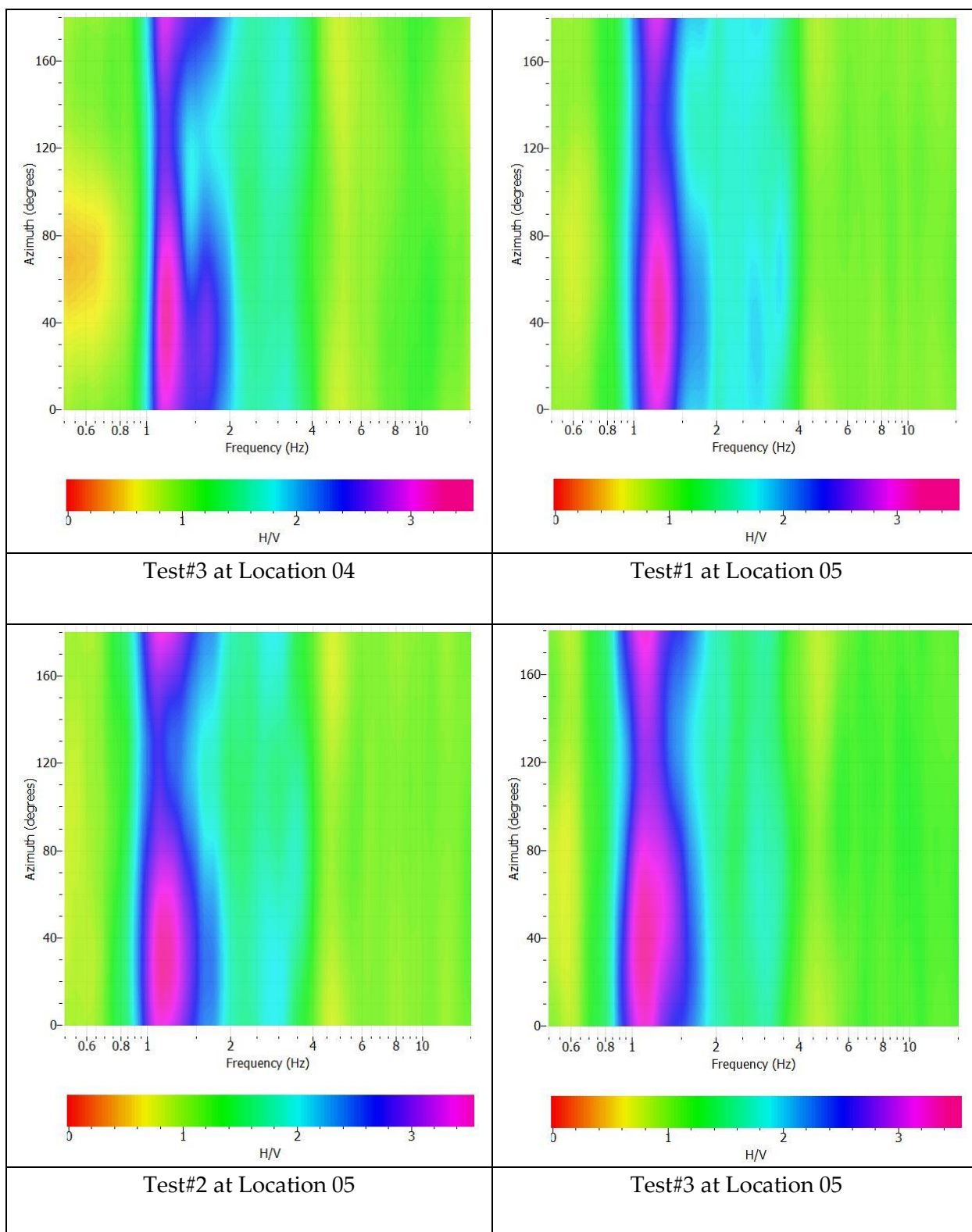

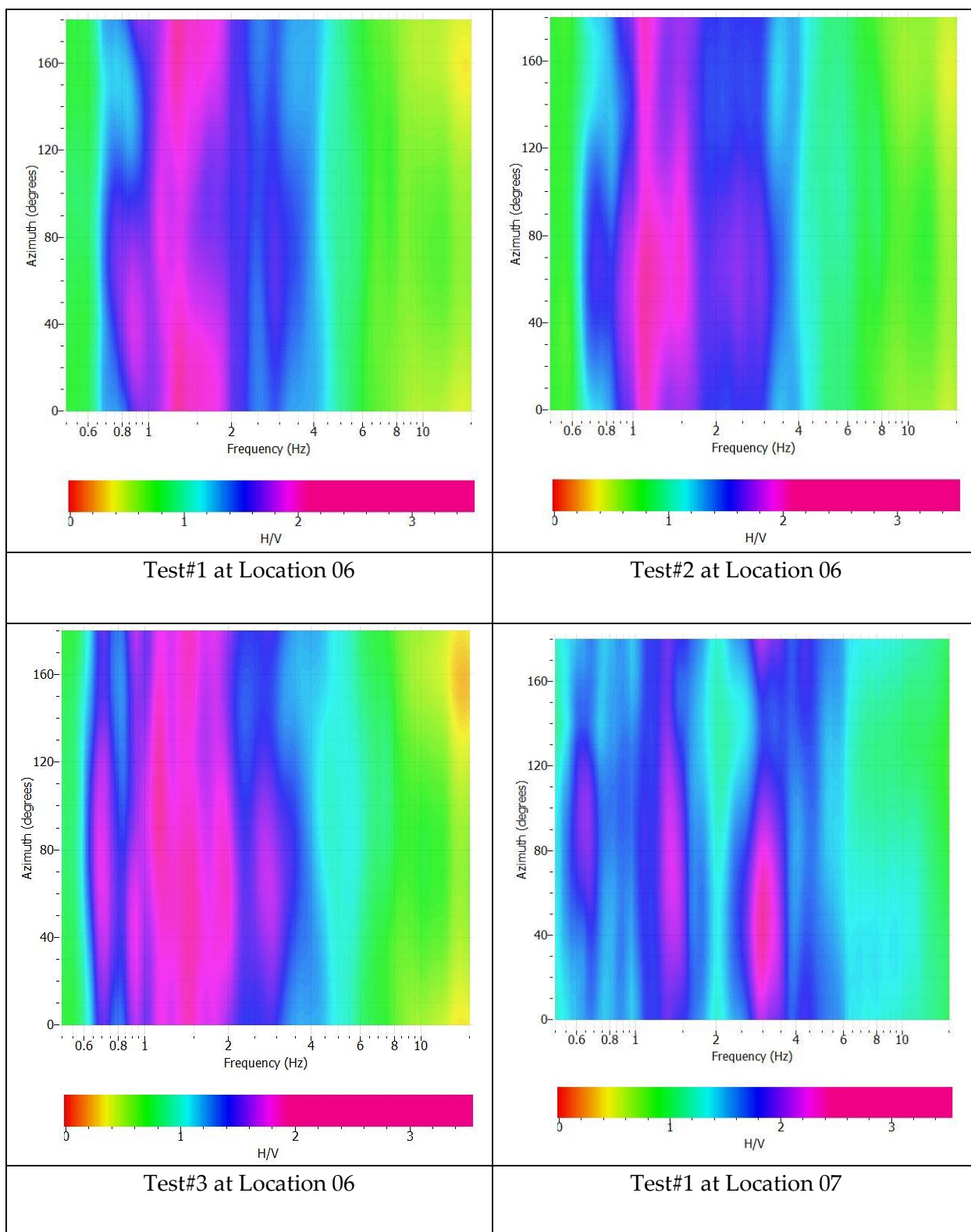

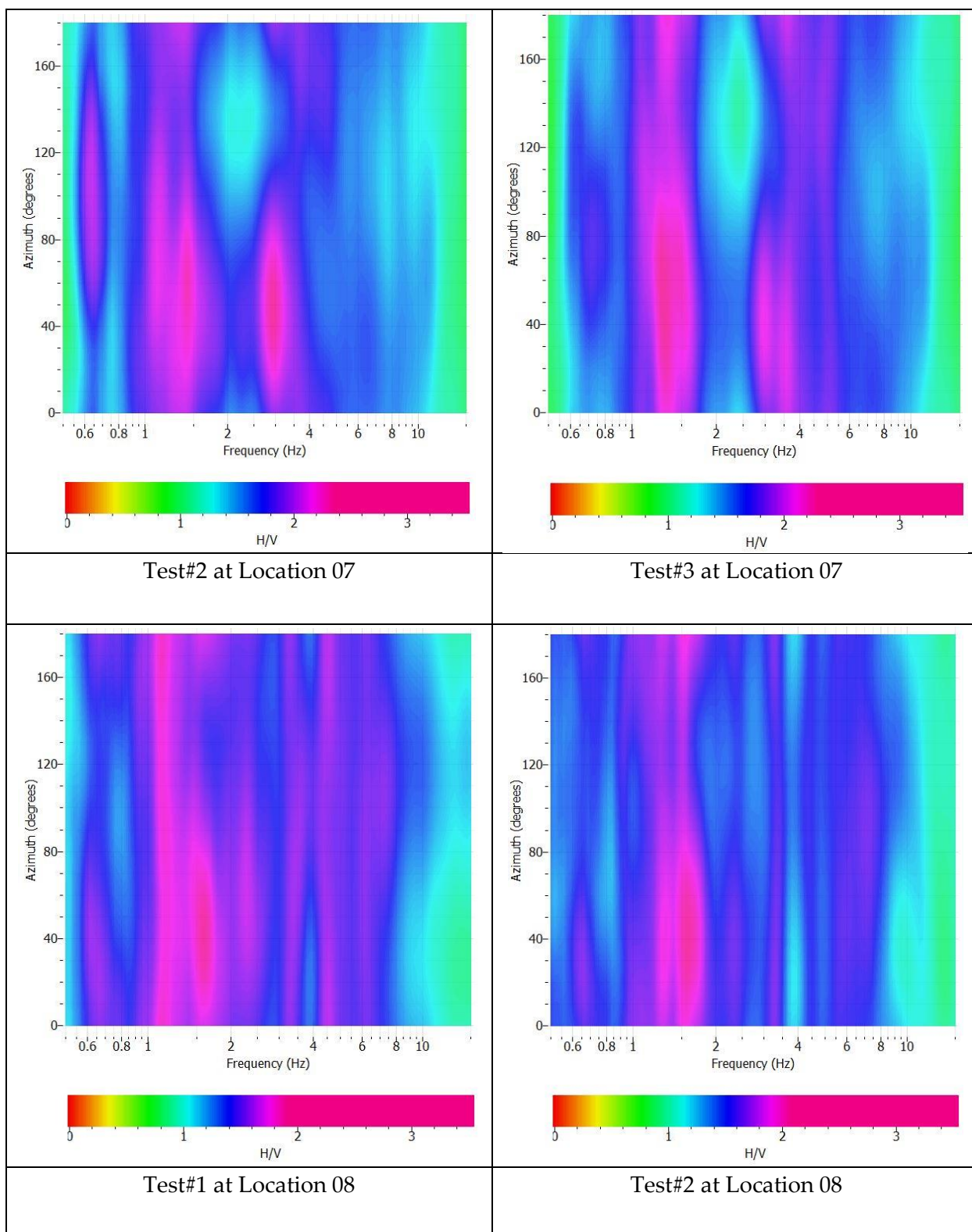

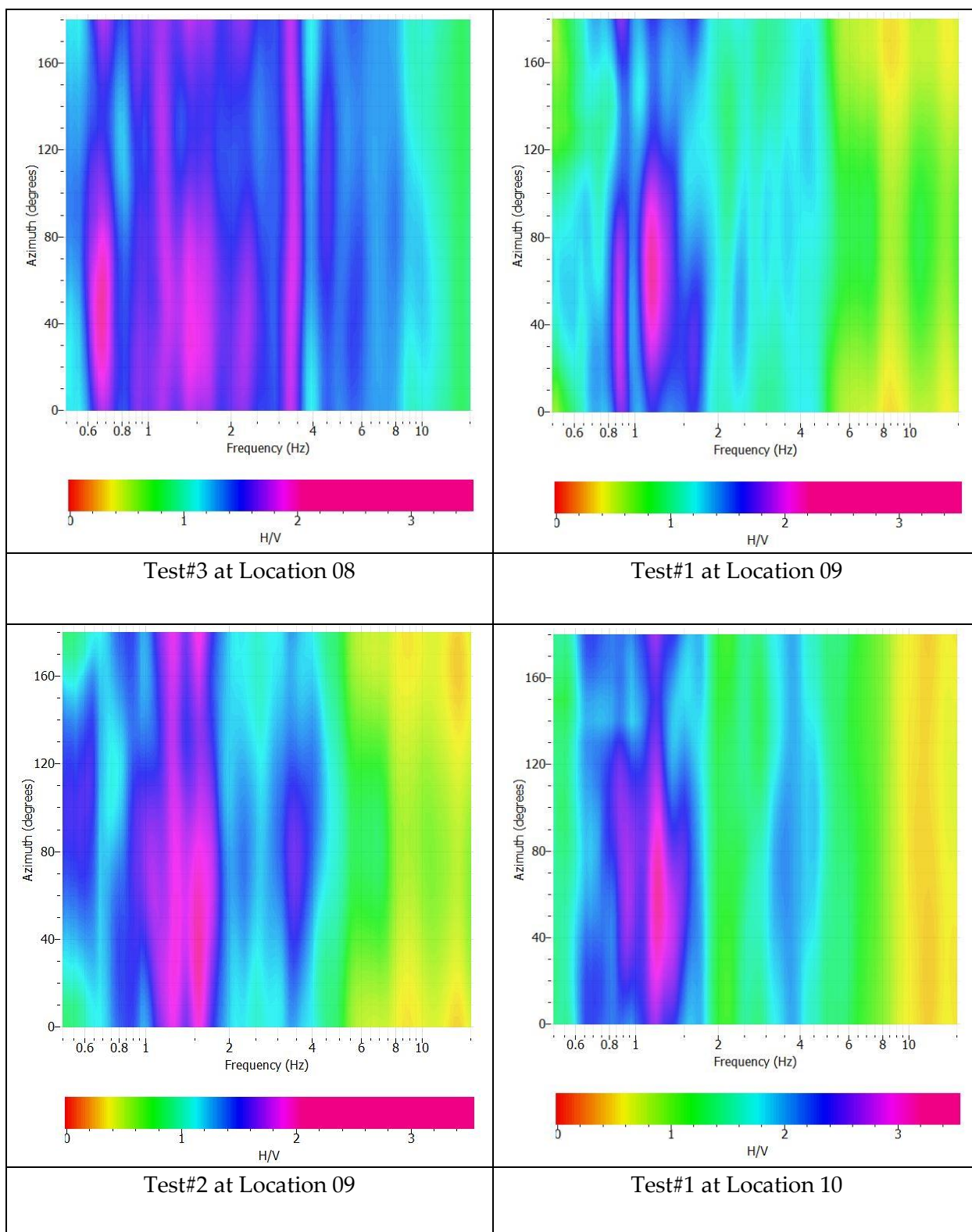

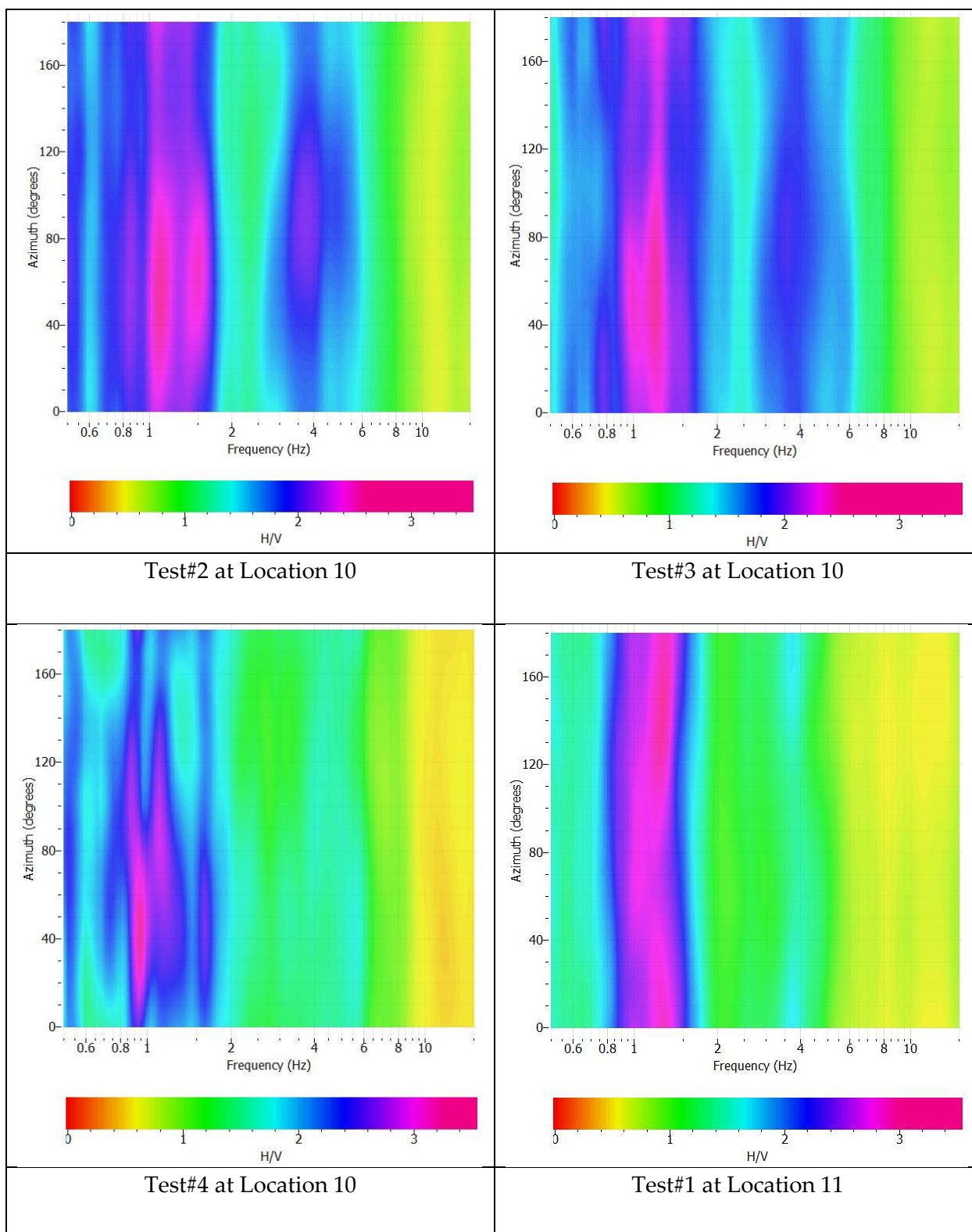

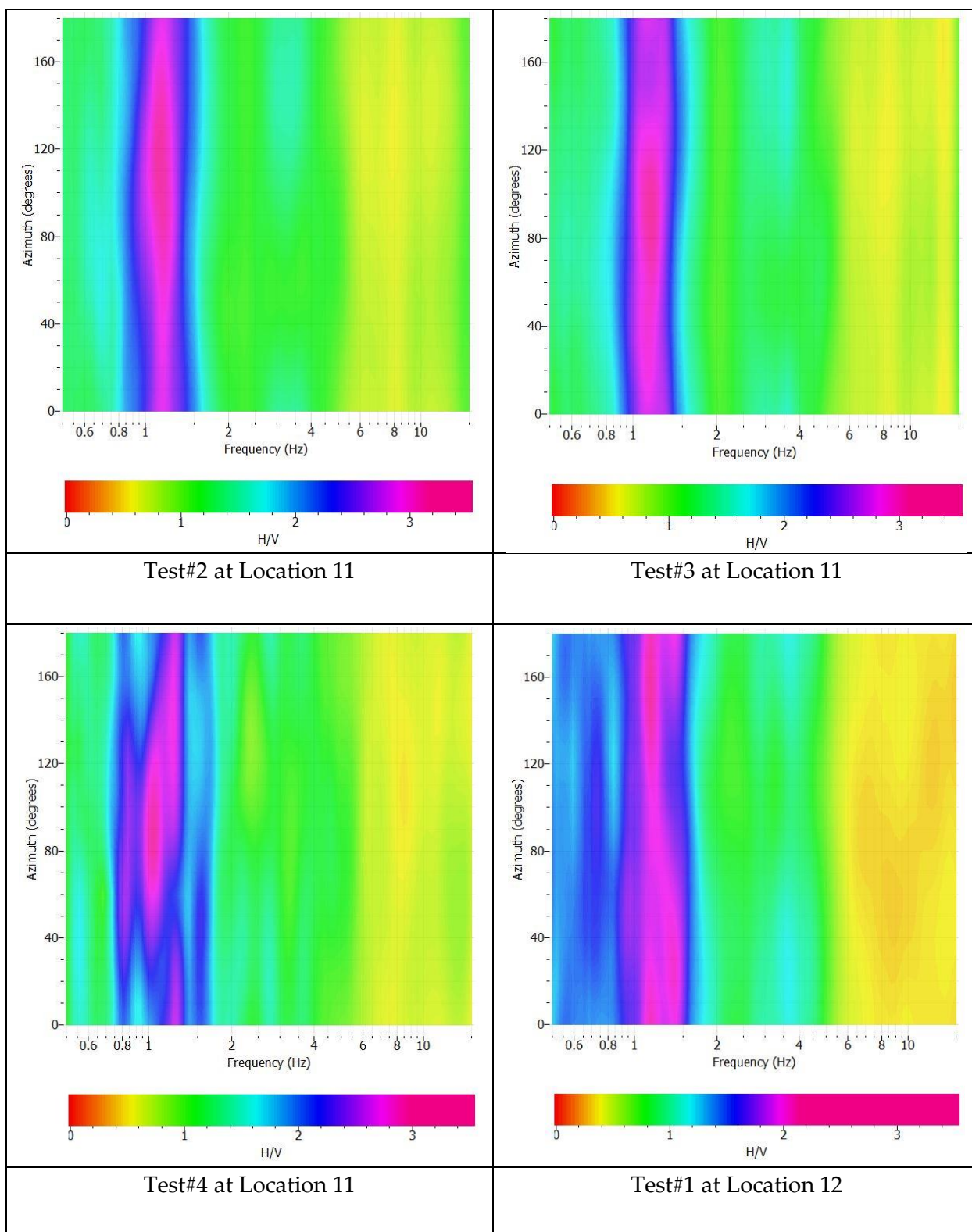

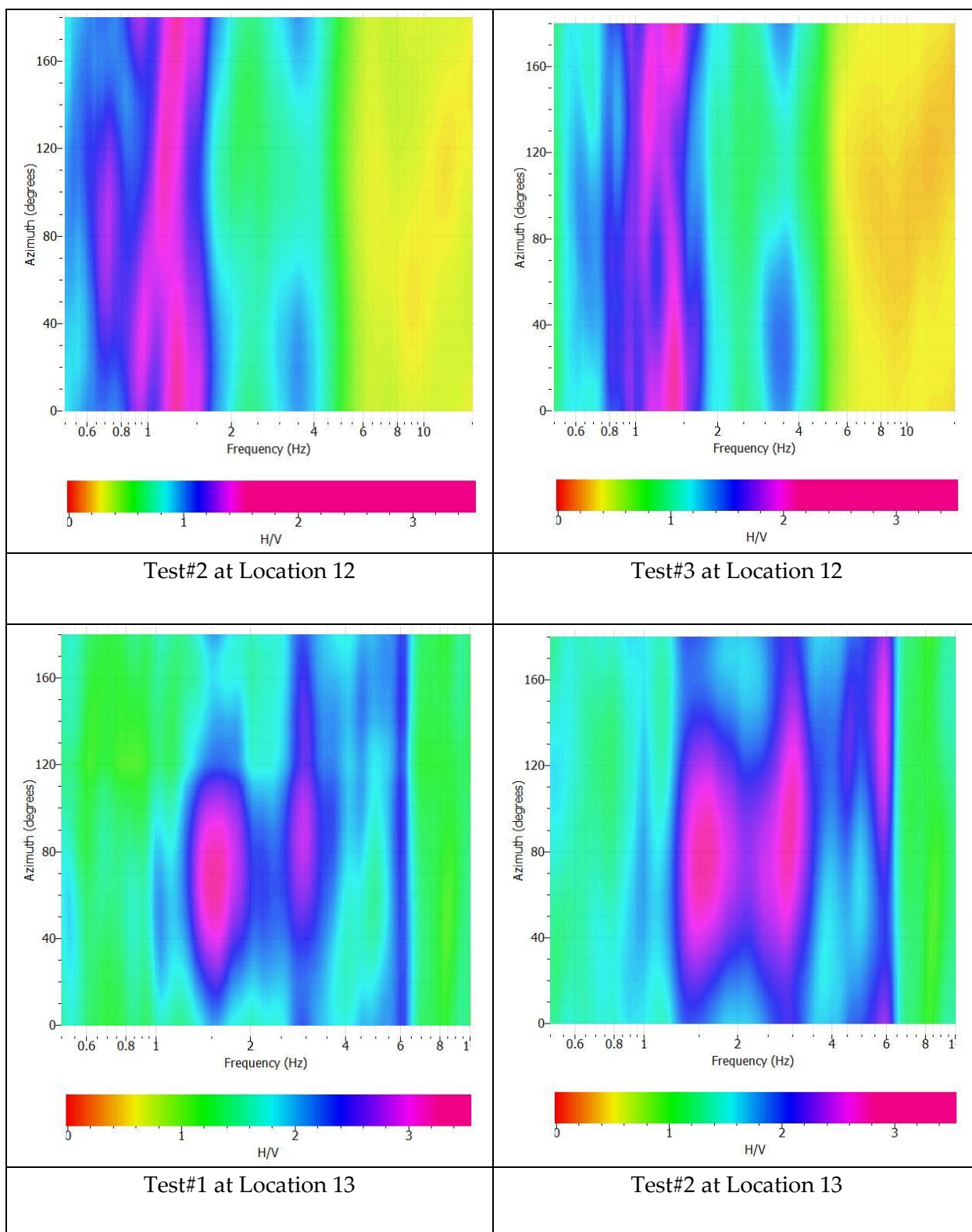

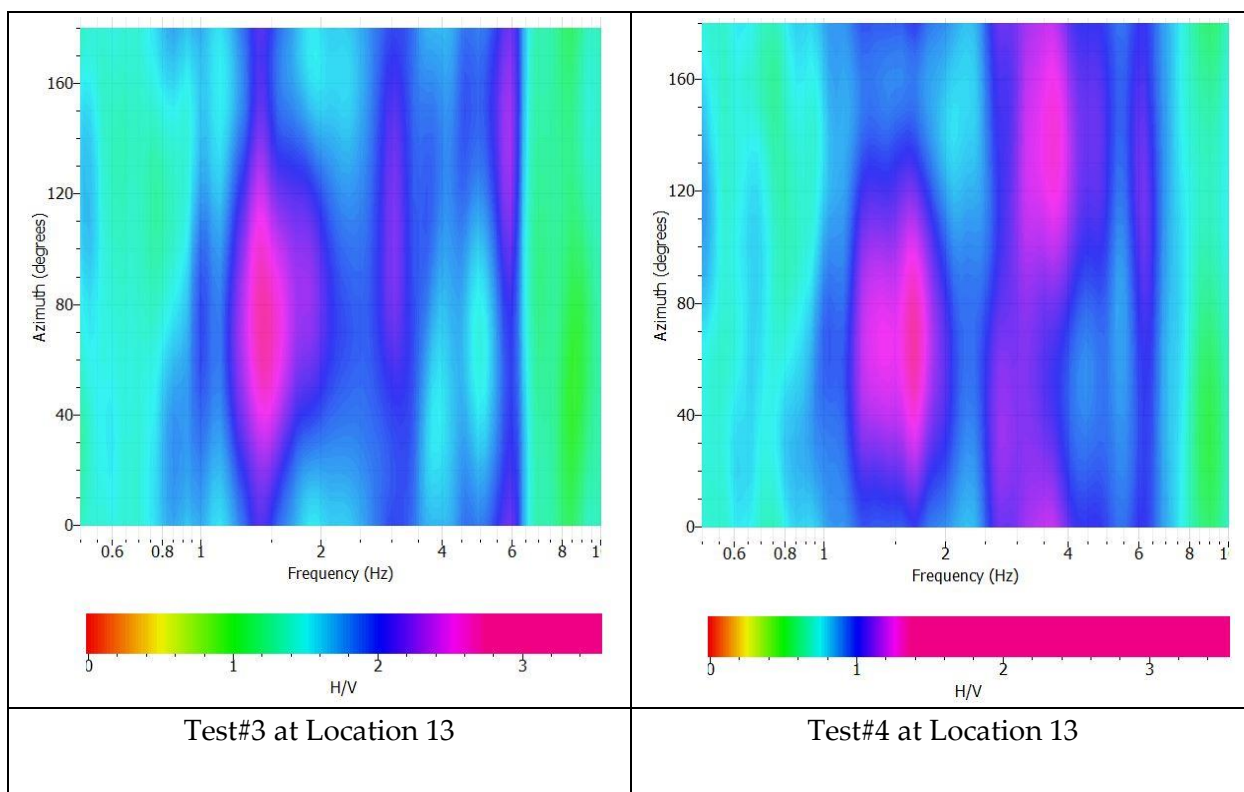

Supplement: Supplementary file 3 [file mmc3.pdf]
